# Insights on the Nutraceutical Properties of Different Specialty Teas Grown and Processed in a German Tea Garden

**DOI:** 10.3390/antiox12111943

**Published:** 2023-10-31

**Authors:** Patricia Carloni, Federico Girolametti, Elisabetta Giorgini, Tiziana Bacchetti, Cristina Truzzi, Silvia Illuminati, Elisabetta Damiani

**Affiliations:** 1 Department of Agricultural, Food and Environmental Sciences-D3A, Università Politecnica delle Marche, Via Brecce Bianche, 60131 Ancona, Italy; p.carloni@univpm.it; 2 Department of Life and Environmental Sciences, Università Politecnica delle Marche, Via Brecce Bianche, 60131 Ancona, Italy; f.girolametti@univpm.it (F.G.); e.giorgini@univpm.it (E.G.); t.bacchetti@univpm.it (T.B.); c.truzzi@univpm.it (C.T.); s.illuminati@univpm.it (S.I.)

**Keywords:** *Camellia sinensis*, single-estate German teas, hot and cold brews, total polyphenol content, antioxidant profile, elemental content analysis

## Abstract

European countries have recently started experimenting with growing and producing their own teas in small quantities, mainly for the specialty tea sector. To characterize European teas, this study investigated a set of five tea types obtained from different *Camellia sinensis* varieties/cultivars, representing various oxidation grades (green, white, yellow, oolong, black), all grown and processed in the only tea garden in Europe (in Germany) that focuses on all five types. Hot and cold brews were studied by measuring the total phenolic (TPC) and flavonoid contents (TFC), the antioxidant capacity and UV-Vis spectra, also with the objective of discriminating between the different tea types and the different plant varieties. The dried leaves were analyzed to measure the content of essential and toxic elements and by ATR-FTIR spectroscopy to determine a chemical fingerprint for identifying the tea varieties and types. The average levels of TPC (hot brew = 5.82 ± 2.06; cold brew = 5.4 ± 2.46 mM GAEq), TFC (hot brew = 0.87 ± 0.309; cold brew = 0.87 ± 0.413 mM CAEq), and antioxidant capacity (ORAC assay-hot brew = 20.9 ± 605; cold brew = 21.8 ± 8.0 mM TXEq, ABTS assay-hot brew = 15.2 ± 5.09; cold brew = 15.1 ± 5.8 mM TXEq, FRAP assay-hot brew = 9.2 ± 3.84; cold brew = 10.4 ± 5.23 mM AAEq) observed compared well with those from other parts of the world such as China, Africa, and Taiwan. The hazard quotient <1 and the hazard index of 0.14 indicate that there is no non-carcinogenic risk from consumption of these teas. The obtained information is essential for elucidating the characteristics and the impact of tea processing and tea variety on the health benefits of these tea products coming from a single European tea garden. This multifaceted approach would help tea growers in Europe increase their knowledge on the health attributes of the teas they grow, ultimately leading to optimization of the nutraceutical properties of these teas.

## 1. Introduction

*Camellia sinensis* (L.) O. Kuntze is a species of plant whose leaves and leaf buds are used to produce tea, one of the most popular non-alcoholic beverages consumed in all corners of the globe. Two major varieties of this species exist: the small-leafed Chinese one, *Camellia sinensis* var. *sinensis*, which originated in South-East China and whose leaves are mainly processed to give green, white, yellow, and oolong teas, and the large-leafed Indian variety, *Camellia sinensis* var. *assamica*, originating in the Assam region and whose leaves are mainly used for processing into black teas [1,2]. A minor, less common variety is *pubilimba*, characterized by dense slivery-haired leaves, mainly processed for green tea by the local people of its growing areas of Guangxi province (China) [3]. Indeed, it is the distinctive processing methods, i.e., extent of oxidation that give rise to the different types of tea mentioned above. The oxidation process is sometimes inappropriately referred to as fermentation, although this is an entirely different process which does not use molecular oxygen for its reactions and where microorganisms (bacteria) are involved [4]. Black tea is a fully oxidized tea where the polyphenols (catechins) are oxidized by polyphenol oxidases to oligomeric theaflavins and polymeric thearubigins which impart the dark colour and aroma of this tea. Green tea instead does not undergo oxidation since the polyphenol oxidases are heat inactivated [5]. Between these two extremes, are placed white and yellow teas which are only mildly oxidized, and oolong teas which are semi-oxidized [6]. 

Globally, the most consumed tea types are black tea and green tea, with black tea accounting for over 70% of the annual tea production followed by green tea accounting for around 20% [7]. India and China are the major producing countries for these two tea types, respectively, followed by Sri Lanka, Japan, Taiwan, and Kenya [8,9], although tea is grown in a smaller scale in over 50 countries throughout different parts of the world from the Americas to Australia [10]. Among these are several European countries that have recently started experimenting with growing and producing their own teas in small quantities, directed mainly for the specialty tea sector. Unlike mass-market or commodity tea, specialty tea is considered a high-grade, organic, loose-leaf tea, usually from small tea gardens, that has been masterfully hand-crafted to deliver a unique flavour profile. In 2016, the “Tea Grown in Europe” Association (EuT) was founded with the aim of promoting an economic activity in this new agricultural sector since parts of Europe (currently 10 countries) are potentially suitable for tea growing [11]. Being in its infancy, there is a lack of scientific data on the nutritional characteristics of European teas, therefore, we recently undertook studies to cover this unexplored tea region. We investigated both the antioxidant properties of hot and cold brews of black, green, and white teas produced across the European territory as well as the content in phytocompounds [12]. In this regard, there is a wealth of scientific evidence showcasing teas’ many health benefits related to the quality and quantity of its phytocompounds [13]. Indeed, the beneficial properties of drinking tea date back to thousands of years, where the Chinese Tang Dynasty considered tea as a medicine able to prevent diseases before their manifestation [14]. We also undertook an elemental analysis study to determine the presence of 15 elements (both potentially toxic and essential) in tea leaves collected from the same tea gardens that participated in the antioxidant study, and to determine the potential risk of exposure to toxic elements from the consumption of European teas [15]. The present study now takes the characterization of European teas a step further, by exploring a set of different types of tea representing various oxidation grades (black, oolong, white, yellow, green) all produced from the same tea garden (Tschanara Tea Garden, Wirtsspezard, Germany) using different *Camellia sinensis* varieties and cultivars. The goal of this tea garden is to optimize the quality of its teas in terms of taste and growth properties, and it is the only tea garden in Europe which primarily focuses on the production of not only green, oolong, and black tea, but also white and yellow tea.

Thus, to characterize the teas produced in this garden, the antioxidants and spectroscopic characteristics of hot and cold brews were studied by measuring the total phenolics and flavonoids content, the antioxidant capacity, and the UV-Vis spectra, also with the objective of discriminating between the different types of tea and the different plant varieties. Furthermore, the dried leaves used to obtain the infusions were analyzed to measure the content of essential and toxic elements and by ATR-FTIR spectroscopy to determine a chemical fingerprint for identifying the tea varieties and types. The obtained information is essential for elucidating the characteristics and the impact of tea processing and tea variety on the health benefits of these tea products coming from a single European tea garden. This multifaceted approach would help tea growers in Europe increase their knowledge on the health attributes of the teas they grow, ultimately leading to optimization of the nutraceutical properties of these teas.

## 2. Materials and Methods

### 2.1. Chemicals and Equipment

All chemicals used were purchased from Merck KGaA (Darmstadt, Germany): [2,2′-azinobis-(3-ethylbenzothiazoline-6-sulfonic acid) diammonium salt] (ABTS), 6-hydroxy-2,5,7,8-tetramethylchroman-2-carboxylic acid (TX), gallic acid (GA), Folin-Ciocalteu reagent (2N solution), potassium persulfate (K_2_S_2_O_8_), sodium carbonate (Na_2_CO_3_), 3′,6′-dihydrosyspiro[isobenzofuran-1[3H],9′[9H]-xanthen]-3-one (fluorescein), [2,2′-azobis(2-methylpropionamidine) dihydrochloride] (AAPH), (+)-Catechin hydrate, sodium nitrite (NaNO_2_), aluminum chloride hexahydrate (AlCl_3_·6H_2_O), sodium hydroxide (NaOH), 2,4,6-tripyridyl s-triazine (TPTZ), iron(III) chloride (FeCl_3_), ascorbic acid (AA), sodium acetate (CH_3_COONa), acetic acid (CH_3_COOH), hydrochloric acid (HCl), potassium dihydrogen phosphate (KH_2_PO_4_), dipotassium hydrogen phosphate (K_2_HPO_4_), ethanol absolute RPE grade. Ultrapure water was generated from a Milli-Q system by Merck Millipore (Merck KGaA, Darmstadt, Germany) and was used for all the experiments. For the tea infusions, mineral water ACQUA SANT’ANNA S.p.A. (Vinadio, Italy) with a fixed residue at 180 °C of 22 mg/L and total hardness of 0.98 °f was used, purchased from the local supermarkets. The mineral composition of this water is reported in Table S1 of the Supplementary.

### 2.2. Tea Samples

**Figure 1:**
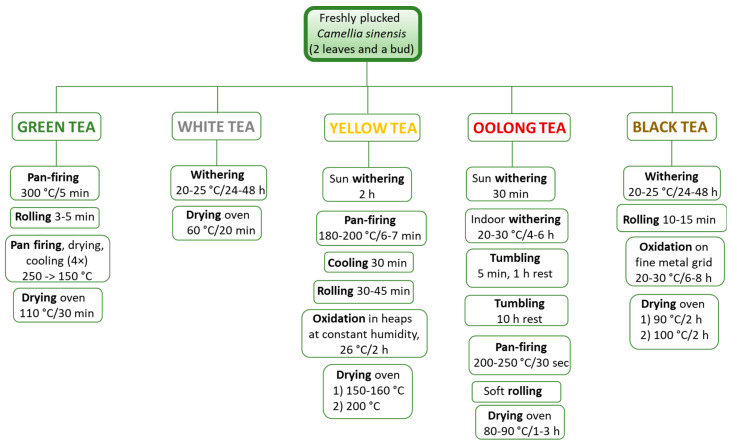
General outline of the different processing steps adopted by Tschanara Tea Garden (Germany) to produce the five tea types.

Eight tea samples were obtained from the Tschanara Tea Garden whose leaves had been harvested in the 2021 season. Apart from 2 tea samples (WV and OA), all the other 6 teas tested in the study were a blend of a minimum of 2 to a maximum of 7 tea batches, all plucked between May and September 2021 from different rows in the same field (BA: 3 batches, BV: 4 batches, BK: 3 batches, GK: 7 batches, YK: 7 batches, OK: 2 batches, OA: 1 batch, WV: 1 batch). The Tschanara Tea Garden is located in Germany, situated on a shielded hillside (213 m above sea level) in Odenthal-Scheuren, in the middle of the Bergische Land and covers an area of 4000 m^2^. Tea has been growing here since 1999, favored by a mild climate, good soil (pH 4.7–5.9) and good drainage. Details regarding the temperature range, growing season, altitude, and average rainfall of this tea garden can be found in the EuT Association 2023 leaflet [11]. The tea samples are described in Table 1 where they are identified with an acronym of two letters indicating the type of tea (W = White; Y = Yellow; G = Green; O = Oolong; B = Black), followed by the country of origin of the cultivar (V = Vietnam; K = Korea; A = Azores). Furthermore, for the brews, a letter indicating the type of brew (C = cold, H = hot) was added at the end when describing the results reported in the tables and figures. A general outline of the steps adopted for processing of the tea leaves to produce the different tea types is reported in Figure 1, which follow the guidelines of the Compendium for tea production in Europe [16].

### 2.3. Preparation of Tea Brews

The tea brews were prepared essentially as described in [12] using 1.0 g of tea leaves and 50 mL of mineral water. Mineral water instead of tap water was used since its composition is stable and known, therefore, it would not be expected to influence the results obtained. Before preparing the infusions, the dry tea samples were all milled utilizing a hand-mill to obtain a homogeneous fine powder for each type of tea. This is essential for reducing the variability in extraction efficiency that could arise from different leaf sizes. Briefly, hot brews were prepared using boiled water (95–100 °C) and an infusion time of 5 min, whereas cold brews were prepared using water at room temperature (20–25 °C), followed by agitation for 5 s and refrigeration (4–6 °C) for 16 h. Both types of brews were then filtered through filter paper (Whatman No. 4), aliquoted, and stored at −20 °C until analyzed. For each tea sample, the two brewing methods were performed in triplicate on three separate days.

### 2.4. Determination of Total Phenolic Content (TPC) 

Total phenolic content in 50 μL of the tea infusions diluted 30× was determined using the Folin–Ciocalteu reagent [17] using the same methodology as described by us [12] and optimized for reading the absorbance at 760 nm on a microplate reader (Synergy HT, Biotek, Winooski, VT, USA). The results are expressed as mM Gallic acid equivalents (mM GAEq), using the linear regression value calculated from the gallic acid calibration curve.

### 2.5. Total Flavonoid Content (TFC)

The total flavonoid content in 50 μL of the tea infusions diluted 10× was determined using a colorimetric assay according to the method of Kim et al. [18] using the same methodology as already described in detail by us [12]. The results are expressed as mM Catechin equivalents (mM CEq) using the linear regression value calculated from the catechin calibration curve. 

### 2.6. Determination of In Vitro Antioxidant Capacity (ABTS, FRAP, ORAC)

A panel of three different assays were used to evaluate the in vitro antioxidant capacity: oxygen radical absorbance capacity (ORAC), ABTS, and ferric reducing antioxidant power (FRAP) assays [19,20,21]. 

The ORAC assay was carried out on 50 μL of tea infusion diluted 800× following the method exactly as recently described by us [12]. The antioxidant capacity is expressed as mM Trolox Equivalents (mM TXEq), using the linear regression value calculated from the Trolox calibration curve. The ABTS assay was performed on 30 μL of each brew previously diluted 120×, following the procedure exactly as described in [12]. For this assay, the antioxidant capacity was determined as inhibition percentage and is expressed as mM Trolox Equivalents (mM TXEq), using the linear regression value calculated from the Trolox calibration curve. The FRAP assay was also performed following the procedure just as it is described in [12] on 50 μL of each brew previously diluted 40×. The results are expressed as mM ascorbic acid equivalents (mM AAEq), using the linear regression value calculated from the ascorbic acid calibration curve.

### 2.7. UV-Vis Spectrophotometric Measurements 

To obtain further information that could help characterize and differentiate the tea brews, spectrophotometric measurements in the UV-visible range were taken [12]. Briefly, 150 μL of each tea infusion diluted 4× was added in each well of a transparent 96-well microplate and the absorbance spectrum (200–500 nm) was recorded in duplicate at constant intervals (Δλ = 2 nm) against water as a blank. The results are expressed as AU (arbitrary units).

### 2.8. ATR-FTIR Measurements and Data Analysis

ATR-FTIR was carried out on a Bruker Invenio-R interferometer equipped with a Platinum ATR accessory mounting a diamond crystal and a Deuterated TriGlycine Sulfate (DTGS) detector (Bruker Optics, Ettlingen, Germany). Tea leaves were crushed in a mortar to obtain a homogeneous powder, which was deposited onto the diamond crystal and gently pressed to obtain a good adhesion to the crystal surface. Then, on this powder, the ATR-FTIR spectrum was collected at room temperature in the 4000–600 cm^−1^ range (128 scans, 4 cm^−1^ spectral resolution). Before each sample acquisition, the spectrum of the background was also collected on the clean diamond crystal under the same conditions. Seven replicates were analyzed for each experimental group. 

Raw spectra were corrected for the contribution of atmospheric CO_2_ and water vapor and vector normalized in the whole spectral range (respectively, atmospheric compensation and vector normalization routines, OPUS 7.5, Bruker Optics, Ettlingen, Germany). Pre-processed spectra were then cropped in the 1800–700 cm^−1^ spectral range, baseline corrected, and submitted to principal component analysis (PCA) with no further pre-processing (Origin PRO 2018 software). For each experimental group, the average spectrum (centroids) together with the average spectrum ± S.D. spectrum was also generated (averaging routine, OPUS 7.5, Brucker Optics, Ettlingen, Germany). 

### 2.9. Elemental Analysis 

Sample preparation and analysis for the determination of elemental content were performed according to Girolametti et al. [15]. Briefly, the treatment was conducted in an ISO 5 clean room laboratory. A specific cleaning procedure with HCl (35.20% Carlo Erba, Milan, Italy, 1:10 *v*/*v*) was adopted for the decontamination of all materials. For the analysis of 14 elements (Ag, Al, As, Cd, Co, Cr, Cu, Fe, Mn, Ni, Pb, Se, V, and Zn), homogenized samples were mineralized with a MARS–6 system (CEM Corporation, Matthews, NC, USA), using 3 mL HNO_3_ and 3 mL H_2_O_2_ per 0.5 g of raw sample, diluted to 10 mL with Milli–Q water (Merck, Darmstadt, Germany) [22]. Determinations were then carried out with a graphite furnace atomic absorption spectroscopy technique (GFAAS, 240Z AA, Agilent Technologies, Santa Clara, CA, USA) [23]. As a carrier gas, argon 5.0 (99.99% pure; Sol S.p.a., Ancona, Italy) was employed. To improve the signal quality for specific elements, the use of a matrix modifier (200 µg L^−1^ Pd in citric acid) was evaluated. Standard solutions were prepared from 1 g L^−1^ stock solution (2–5% HNO_3_, Carlo Erba, Milan, Italy). The calibration curve method was applied for the elemental quantification. The determination of Hg was carried out on 0.1 g of raw sample by a thermal decomposition amalgamation atomic absorption spectrometry (TDA AAS) technique using a direct mercury analyzer (DMA−1, FKV, Milestone, Sorisole, Italy). All analyses were performed in triplicate (*n* = 3). Information on the method’s linearity, the instrumental LOD, and LOQ assessed for the studied matrix are reported in Girolametti et al. [15]. DORM–2 and 1648a (Dogfish Muscle CRM and Urban Particulate Matter SRM, National Research Council of Canada, Ottawa, ON, Canada) were used to evaluate the analytical methodology’s accuracy.

### 2.10. Health Hazard Estimation

Human safety from the consumption of tea prepared from these tea leaves was evaluated by comparing the concentrations obtained with the limits imposed by the various National Regulations. In addition, the hazard quotient (HQ) was determined following Girolametti et al. [15] to assess the potential non-carcinogenic health risk associated with element exposure through tea drinking:

HQ = ADD/RfD

ADD = C × IR/BW

where ADD is the daily intake dose and RfD is the corresponding daily intake reference dose. C (mg kg^−1^) is the mean concentration of the element in tea leaves, IR is the average tea consumption by Europeans (23.48 g person^−1^ day^−1^), BW is the average body weight of a European adult (70 kg), and RfD (mg kg^−1^ day^−1^) is the daily intake reference dose suggested by the United States Environmental Protection Agency (USA EPA) or World Health Organization (Joint FAO/WHO Expert Committee on Food Additives). Since children rarely have the habit of drinking tea, the health risk assessment was only performed for adults. A value of less than 1 assumes an absence of risk due to the intake of a single element by consumers. In addition, to assess the simultaneous effect of exposure to all elements considered, the hazard index (HI) was measured as follows:

HI = HQ_1_ + HQ_2_ + HQ_3_ + … + HQ_n_

A value of less than 1 indicates a safe condition against total exposure to the different elements.

### 2.11. Statistical Analysis 

The results of TPC, TFC, and antioxidant assays are expressed as mean values with standard deviation (SD) from at least three independent experiments each performed on the infusions prepared independently three times (*n* = 9). Statistical differences were obtained through an analysis of variance (ANOVA), followed by Tukey’s multiple comparison test at a 95% confidence level (*p* ≤ 0.05). The results of UV-Vis spectrophotometric measurements mediated for each tea infusion were processed using multivariate chemometric techniques involving principal component analysis (PCA) together with TPC, TFC, and antioxidant activity data using XLSTAT software (version 2017.1.1, Addinsoft SARL, Paris, France).

For elemental content, each analysis was performed in triplicate. The results are expressed as mg kg^−1^, mean ± standard deviation (min–max). Statistical analyses were performed using the RStudio software (R version 4.2.2) and the “ggplot2” package. Sample groups were compared using a one-way analysis of variance (ANOVA), followed by the Tukey’s test, at the 95% confidence level.

For ATR-FTIR, normally distributed data are presented as mean ± SD. Significant differences between groups were determined by a one-way ANOVA followed by a Tukey multiple comparison (software package GraphPad Prism 6.0, San Diego, CA, USA). Statistical significance was set at *p* < 0.05 (*p* > 0.05, n.s.; *p* < 0.05, *; *p* < 0.01, **; *p* < 0.001, ***; and *p* < 0.0001, ****).

## 3. Results

### 3.1. Total Polyphenol and Flavonoid Contents in the Tea Brews 

**Figure 2:**
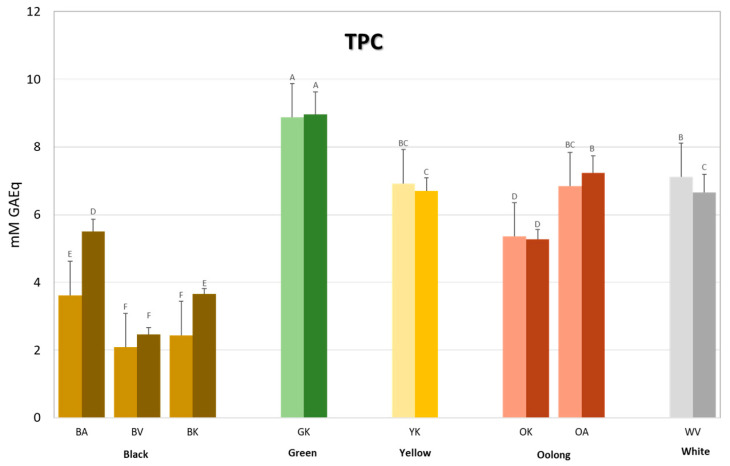
Total polyphenol content (TPC) of the tea brews measured using Folin–Ciocalteu’s reagent. Bars are coloured according to the type of tea (brown = black tea; green = green tea; yellow = yellow tea; rust = oolong tea; grey = white tea) and to the type of brew (light shade = cold brew; dark shade = hot brew). Letters above the bars indicate homogeneous sub-classes resulting from Tukey’s post hoc multiple comparison test (*p* < 0.05). Cultivars are described by the second letter in the Code: A = Azores; V = Vietnam; K = Korea.

For the tea brews obtained from the five different tea types, both the total polyphenol content (TPC) and total flavonoid content (TFC) were determined, since the latter represents the major class of polyphenols found in *Camellia sinensis*. The results in TPC for both hot (dark shade) and cold (light shade) brews are reported in Figure 2 and Table 2. Regardless of the cultivar/variety and type of brew, from Figure 2 it is evident that the green tea has the highest TPC of all the tea types. It has on average a 2–3-fold higher content than the black teas (Hot brews = 9.0 vs. 3.9 mM GAEq; Cold brews = 8.9 vs. 2.7 mM GAEq), reflecting the degree of oxidation. In general, the degree of oxidation is also mirrored by the TPC of the other tea types, where the white and yellow teas, which are mildly oxidized, have a higher TPC than the semi-oxidized oolong teas, and where all three tea types display a higher TPC than the fully oxidized black teas. Regarding the differences between cultivars/variety, which is only valid for the black and oolong teas, it appears that the *assamica* variety, Azores cultivar (BA and OA), is endowed with significantly higher TPC than the *sinensis* variety, Korean cultivar (BK and OK), when prepared either as a hot brew or as cold one. For the black teas, the *pubilimba* variety, Vietnam cultivar, is more similar to the *sinensis* variety, where the only significant difference was observed in the hot BK brew which had a higher TPC (BKH = 3.7 vs. BVH = 2.5, BVC = 2.1, BKC = 2.4 mM GAEq). Concerning the type of brew, the only significant differences were observed in the black teas and in the white tea but with an opposite trend. For the black teas, a significantly higher TPC was observed, as already mentioned, for the *sinensis* variety (BK), but also for the *assamica* one (BAH = 5.5 vs. BAC = 3.6 mM GAEq). Whereas for the white tea, a higher TPC was noted in the cold brew compared to the hot one (WVC = 7.1 vs WVH = 6.7 mM GAE). For the green, yellow, and oolong teas, no significant differences were observed among the types of brews.

With regards to TFC, the results reported in Table 2 follow exactly the same trend as those of TPC as would be predicted being flavonoids, in particular the sub-class of flavanols, the most abundant class of polyphenols found in tea [24]. These observations are supported by the significant correlation between TPC and TFC obtained using Pearson’s correlation coefficient reported in Table 3. However, it is worth noting that there are more significant differences between the hot and cold brews compared to TPC. Indeed, higher TFC was observed in cold brews of both the green tea and the oolong tea OA, compared to their hot-brewed counterparts, whereas the same significant differences between the hot and cold brews for the black and white teas were still present. 

### 3.2. Antioxidant Capacity of the Tea Brews

To obtain an overall depiction of the potential health benefits of the German teas in terms of antioxidant capacity, a panel of three independent and well-recognized assays were used which are based on slightly different principles [25]. The ORAC assay is a direct competition method which mainly relies on hydrogen atom transfer (HAT) mechanism, whereas the ABTS and FRAP assays are considered indirect methods, where the underlying mechanisms are based on HAT and single-electron transfer (SET) for the ABTS assay and SET for the FRAP assay [26].

**Figure 3:**
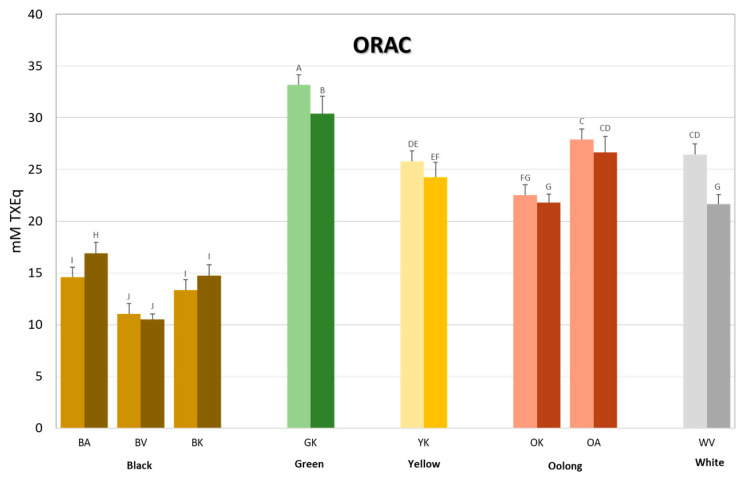
Antioxidant activity of the tea brews measured with the ORAC assay. Bars are coloured according to the type of tea (brown = black tea; green = green tea; yellow = yellow tea; rust = oolong tea; grey = white tea) and to the type of brew (light shade = cold brew; dark shade = hot brew). Letters above the bars indicate homogeneous sub-classes resulting from Tukey’s post hoc multiple comparison test (*p* < 0.05).

The results obtained using the ORAC assay are reported in Figure 3, whereas those obtained using the other two assays are reported in Table 2. Knowing that polyphenols elicit strong antioxidant properties, the results reported in Figure 3 are as expected, since they reflect the TPC content measured in the teas, corroborated also by the high correlation coefficient (Table 3). The antioxidant capacity follows, in general, the extent of oxidation, with the highest antioxidant capacity measured in the unoxidized green tea, and the lowest in the oxidized black teas, and with the other mild and semi-oxidized teas falling in between. Concerning the differences between the two brewing methods, significant findings were observed in only one black tea, BA, where the hot brew had a higher antioxidant capacity than the cold one (16.9 vs. 14.6 mM TXEq), and in the green and white teas, where the cold brew appeared to have a significantly higher antioxidant capacity than the hot one (GKC = 33.2 vs. GKH = 30.4 mM TXEq; WVC = 26.5 vs. WVH = 21.7). For the other tea types, no statistical differences were observed between the two brewing methods. 

Upon using the other two assays (ABTS and FRAP), the results obtained and reported in Table 2 follow a very similar trend with regards to antioxidant profile as the ORAC assay, despite being both based on indirect methods but with different mechanisms for determining antioxidant capacity. Indeed, the results obtained using these two assays strongly correlate with those from the ORAC assay, TPC and TFC. However, with regards to the brewing method, the FRAP assay was able to show more differences among the two brewing techniques. In fact, in all cases, with the exception of the black teas, the antioxidant capacity was always significantly higher in the cold brew. Instead for the black teas, no differences were observed amongst the hot and cold brews. 

### 3.3. UV-Visible Spectral Characteristics of Tea Brews 

The UV-Vis spectra of the samples are reported in the Supplementary (Table S2). Two absorption bands can be detected in the ranges from 220 to 240 nm and from 260 to 300 nm, and another broad absorption band appears around 300–400 nm. These bands can be related to phenolic compounds present in the tea infusions. In addition, caffeine shows a maximum absorption at around 275 nm, so the spectrum in this region could also be related to the presence of this compound [27]. On closer analysis (Figure S1) of the spectra of the different tea types produced from the *sinensis* variety (Korea), the intensity of absorption around 226 nm appears to be strongly related to the type of tea and diminishes with the degree of oxidation; furthermore, the broad absorbance between 300 and 440 nm seems to follow a different pattern. These observations prompted us to check a possible differentiation of tea types according to their UV-Vis spectra and, with the aim of confirming these tendencies, a PCA analysis was performed using the entire spectral range together with total phenols and flavonoids and antioxidant activity data.

### 3.4. Principal Component Analysis (PCA) on Tea Brews

Multivariate analysis was applied on data obtained from the analysis of cold tea brews to understand if these variables can be used to differentiate the samples according to the type of tea or to the type of variety/cultivar used. With this aim, the spectral data obtained from the UV-Vis analysis between 200 and 500 nm of the cold tea brews were statistically elaborated together with the results obtained on the quantification of total phenols, total flavonoids, and antioxidant activity assays using a PCA analysis for the reduction of the data dimension and visualisation of similarities and differences among samples.

The PCA model that was used led to five significant principal components (PC) with an eigenvalue >1 that explained the 99% of the total system variability as shown in the Supplementary (Table S3) where the eigenvalues and the variance explained are reported.

The first two coordinates describe most of the total variability (87.5%): PC1 (46.8%) includes most of the information deriving from the antioxidant tests (FOLIN, FRAP, ABTS, ORAC, FRAP), together with the contributions of the absorption between 220 and 300 nm; absorptions between 200 and 220 nm, and between 300 and 400 nm were instead mainly comprised in PC2 (40.7%), showing that all the variables described by the UV-Vis spectra contribute to the differentiation of the teas.

**Figure 4:**
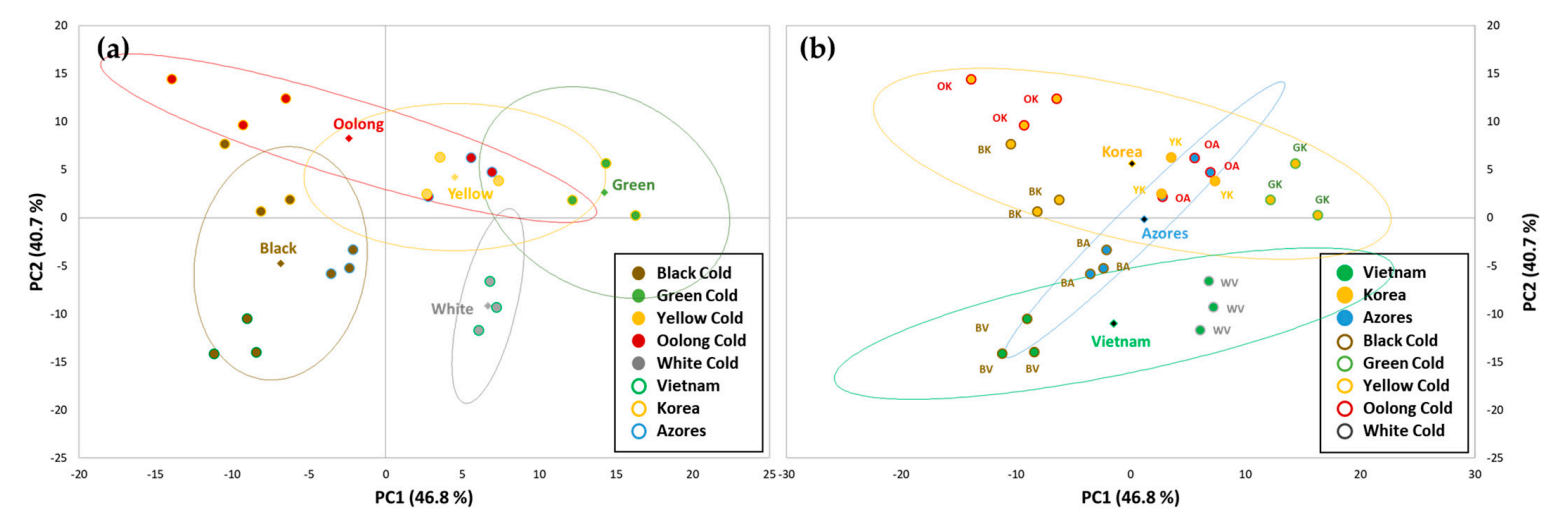
Score plot of the first two components obtained by PCA performed with data obtained from total phenol (TPC) and flavonoid (TFC) content, antioxidant activity (ORAC, ABTS, FRAP), and UV-Vis absorption analysis from 200 to 500 nm of the cold tea brews. The type of tea (**a**) and the cultivar (**b**) were used as passive variables to represent confidence ellipses corresponding to the 80% confidence interval. The samples are coloured depending on the passive variable (filled dot).

Using the tea type as passive variable to represent confidence ellipses corresponding to the 80% confidence interval enclosing teas of the same type, four well-differentiated groups of teas (black, oolong, green, and white) are obtained, while yellow tea shows scores very similar to those of oolong teas (Figure 4a). A similar analysis performed using the tea cultivar as passive variable shows that teas obtained from the Korean cultivar (var. *sinensis*) are well differentiated from samples produced from the Vietnam cultivar (var. *pubilimba*) while for tea produced from the Azores cultivar (var. *assamica*), no clear differentiation of these tea brews can be observed (Figure 4b).

### 3.5. ATR-FTIR Analysis on Tea Leaves 

**Figure 5:**
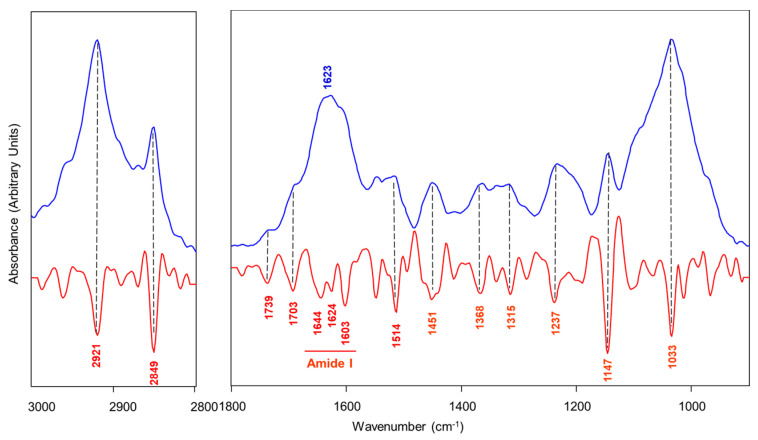
Representative IR spectrum of tea leaves from Tschanara Tea Garden. The spectrum is displayed in the 3000–2800 cm^−1^ and 1800–900 cm^−1^ ranges both in absorbance (blue line) and second derivative (red line) modes, to better identify the most significant peaks which are marked together with the position (wavenumbers).

In this study, the eight teas were also analyzed by ATR-FTIR spectroscopy to determine a chemical fingerprint that would help identify the teas according to the type (processing method) and variety/cultivar. In Figure 5, the IR spectrum representative of the tea leaves is shown. The spectrum is displayed in the 3000–2800 cm^−1^ and 1800–900 cm^−1^ ranges both in absorbance (blue line) and second derivative mode (red line); the latter is to better identify the most significant peaks which are displayed as minima. The peaks assignments, reported in Table 4, was performed according to the literature data [28,29]. The water content present in the leaves was irrelevant since the analysis was performed on dry tea leaves. As confirmation of this, the typical peak at ~2130 cm^−1^ due to the combination band of water is absent in all spectra of the teas studied, hence the choice of showing only the two regions (3000–2800 cm^−1^ and 1800–900 cm^−1^) with the most significant peaks (Figure 5).

**Figure 6:**
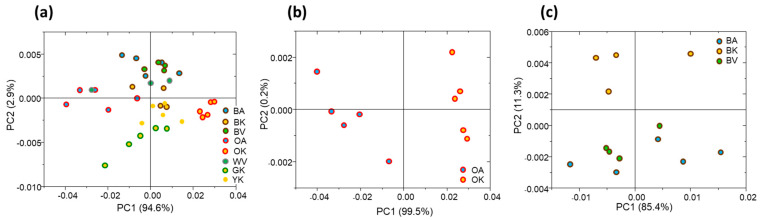
PCA of ATR-FTIR spectra of all tea leaves (**a**), oolong teas (**b**), and black teas (**c**). The analysis was performed in the 1800–700 cm^−1^ spectral range.

The spectral data of all the tea leaves were submitted to principal component analysis. The analysis of the PCA scores plot evidenced only a weak differentiation in the spectral profiles as regards both tea types and cultivars (Figure 6a); in fact, only the oolong teas, OA and OK, and the green one from Korea (GK) were partially separated from all the others. For a deeper analysis, the pairwise PCA of oolong tea leaves was performed (Figure 6b), displaying a complete segregation between the two varieties/cultivars (PC1 axis, explained variance 99.5%). Finally, regarding the black tea leaves (Figure 6c), a strong separation was observed between all three varieties/cultivars (PC2 axis, explained variance 11.3%). 

### 3.6. Essential and Potentially Toxic Elements 

The comprehensive datasets of the elemental content for essential and potentially toxic elements in the tea leaves from the German tea garden are provided in Tables S4 and S5, respectively. 

**Figure 7:**
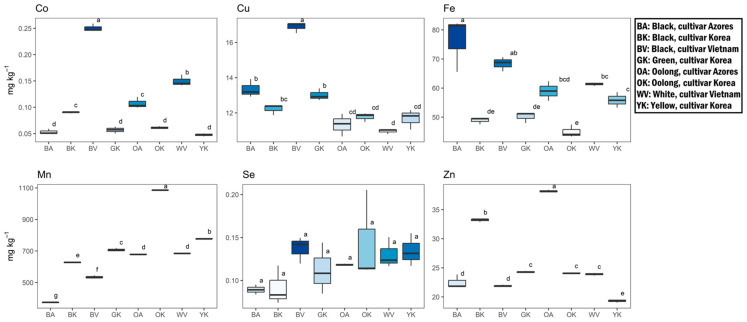
Essential elements content (mg kg^−1^) in differently processed tea leaves from the Tschanara Tea Garden. Different letters indicate statistically significant differences (*p* < 0.05) between different tea types.

**Figure 8:**
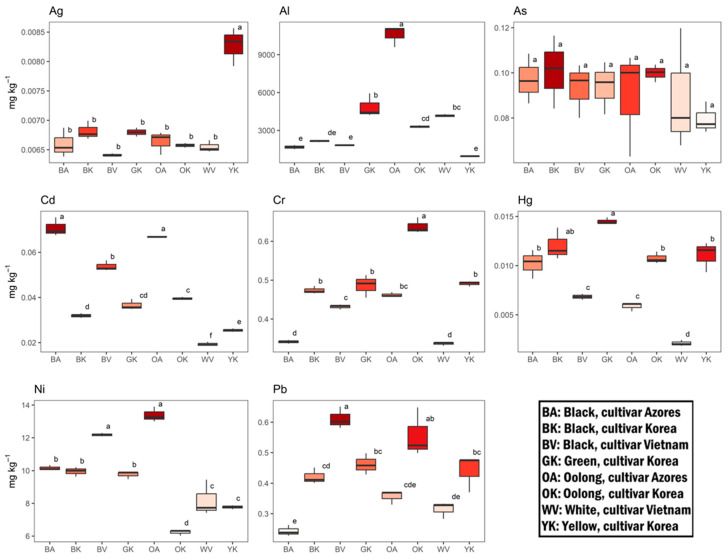
Potentially toxic elements content (mg kg^−1^) in differently processed tea leaves from Tschanara Tea Garden. Different letters indicate a statistically significant difference (*p* < 0.05) between different tea types.

The average levels of essential elements were arranged according to the following order: Mn > Fe > Zn > Cu > Se > Co (Figure 7). V was always below the instrumental limit of detection (LOD). Mn, Fe, and Zn were the major elements (684 ± 204, 58 ± 11, and 26 ± 6 mg kg^−1^, respectively), accounting for 98.4% of the total essential elements analyzed. Oolong tea of Korean cultivar (OK) showed the highest level in Mn (1086 ± 2 mg kg^−1^). With the exception of Se, all samples showed statistically significant differences (*p* < 0.05) in the elemental distribution. However, there was not a distinct pattern showing a comprehensive higher concentration of essential elements in one type of tea rather than another. The potentially toxic elements’ concentrations followed the order of Al > Ni > Cr > Pb > As > Cd > Hg > Ag (Figure 8). The Al fraction significantly dominated the others, accounting for 99.4% of the total. An extremely high concentration was recorded in the Oolong tea from the Azores cultivar (OA) (10.5 ± 0.9 g kg^−1^). 

For the other elements, the concentrations showed a high variability depending on the type of tea, but as for the essential elements, no consistent trend could be identified. The only exception was As, the content of which was statistically similar (*p* > 0.05) in all samples. 

**Figure 9:**
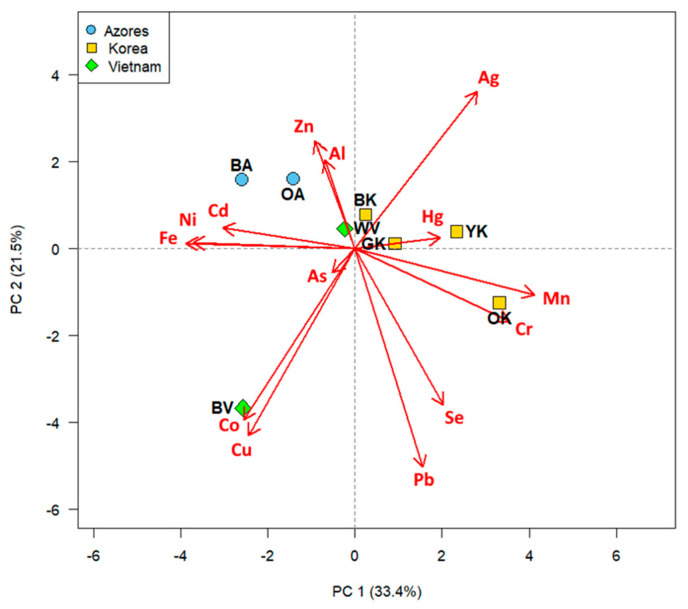
2D Biplot of PC1 vs PC2 performed on the data of essential and potentially toxic elements found in differently processed tea leaves from Tschanara Tea Garden.

Multivariate principal component analysis (PCA) performed on the data of essential and potentially toxic elements, extracted four principal components accounting for 86% of the total variance (Table S6). The PC1 vs PC2 biplot (54.9% of the total variance, Figure 9) resulted in a clear clustering of samples according to the variety/cultivar of origin. In particular, the *sinensis* variety, Korean cultivar (PC1, positive scores) was associated with higher levels of Hg, Mn, and Cr. The *assamica* variety, Azores cultivar, on the other hand, was in the negative PC1 scores and was associated with higher Al, Zn, Cd, Fe, and Ni contents. The *pubilimba* variety, Vietnamese cultivar (BV) was found associated with higher Co and Cu concentrations in the negative PC1 and PC2 axis. Finally, the white tea (WV) from the same variety and cultivar as BV did not appear to be associated with any element. From this analysis, the treatment that the tea leaf undergoes to produce the different tea types did not appear to affect the distribution of the elements.

In order to find potential relationships between the contents of the elements, a correlation analysis was also carried out (Figure S2). This analysis revealed pairs of positively correlated elements such as Cr-Mn (*p* = 0.008172, r = 0.8455813) and Al-Zn (*p* = 0.031, r = 0.7531089) and pairs of negatively correlated elements such as Cr-Fe (*p* = 0.01772, r = −0.7975482), Mn-Fe (*p* = 0.01255, r = −0.8206391), and Ag-As (*p* = 0.04322, r = −0.7218066).

#### Exposure Risk Estimation

Currently, there has been no adoption of a European regulation regarding limits on elemental content in tea leaves. A comparison between the obtained concentrations and the limit values set by the various National Regulations [15] showed that the content of potentially toxic elements was lower in almost all cases. The exceptions were Cu with regard to the extremely restrictive Canadian limit of 2 mg kg^−1^ and Ni concerning the Indian limit value of 5 mg kg^−1^. It is important to note that these values are extremely heterogeneous as each country sets a different threshold in accordance with its own National Regulation. 

**Figure 10:**
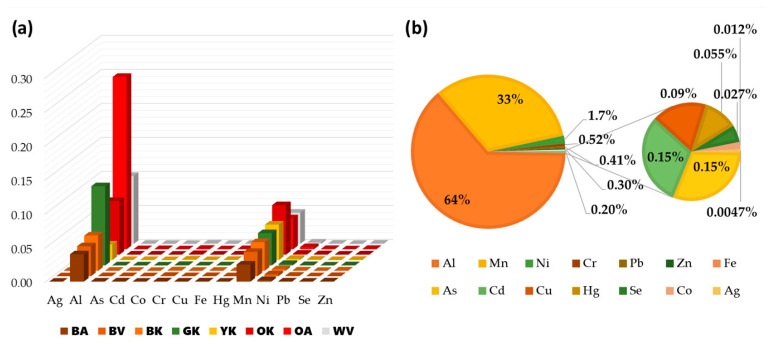
Elemental-related hazard quotients (HQs) associated with differently processed tea leaves from Tschanara Tea Garden (**a**), and average elemental-related hazard quotients (HQs) percentage (**b**).

The measured HQs showed values in the following order Al > Mn > Ni > Cr > Pb > Zn > Fe > As > Cd > Cu > Hg > Se > Co > Ag (Table S7). All HQ values did not exceed 1, indicating that there is no carcinogenic risk from consumption of this product (Figure 10a). The measured mean value of the hazard index (HI) was 0.14, suggesting that the effect resulting from synergistic exposure to the considered elements is not significant enough to pose a non-carcinogenic risk to European consumers. However, the results suggested that Al and Mn are the most important contributors, accounting for 97% of the total HI (Figure 10b). 

## 4. Discussion

European tea is still in an experimental phase with several small projects scattered in different countries, with the Azorean islands having the longest tea producing tradition [30]. The teas grown in Europe defy many of the traditional procedures of tea cultivation, breaking the accepted conventions for tea growing, yet they are able to offer superb quality and are endowed with nutraceutical properties comparable to those from other parts of the world [12]. Another interesting aspect is that each of the tea projects underway is not bound to the traditions of a certain style of tea making, therefore, each garden has the possibility to develop its own tea style. Indeed, this is exemplified by the teas produced in Germany in Tschanara Tea Garden, the object of the present study.

For making the different tea types, the tea garden is experimenting with three tea varieties, the more common *Camellia sinensis* var. *sinensis* and *assamica*, but also with the less common *pubilimba* one, and with selected cultivars (from Korea, Vietnam, and Azores) that have higher frost resistance. The goal is to optimize the quality of teas in terms of taste and growth properties. From the results of the present study, another dimension can now be added to the characterization of these teas: nutraceutical properties and the presence of essential and potentially toxic elements. Firstly, the results undeniably demonstrate that the degree of oxidation of the tea leaves affects the polyphenol and flavonoid contents and, consequently, the antioxidant capacity. This should be reassuring to any tea farmer knowing that whatever personalized procedure is used for obtaining the different tea types, is confirmed by the nutraceutical properties observed in the tea brew. It is in fact well known that green tea is endowed with greater antioxidant activity than fully oxidized black or semi-/partially oxidized oolong, white, and yellow teas as observed by us and others [4,31,32]. Unoxidized teas maintain higher levels of catechins, the main bioactive constituents (they account for about 80% of the total polyphenolic content of tea), than those that undergo any degree of oxidation, as during this process, the simple catechins oxidize and dimerize/polymerize to yield complex quinonic structures, theaflavins and thearubigins, which still possess antioxidant activity [33]. The PCA performed on the tea brews using the biochemical assays data combined with UV-Vis spectroscopy data, also confirmed the variations among the tea types as four distinct groups were obtained (black, oolong, green, and white); the yellow tea was similar to the oolong teas. The levels of TPC, TFC, and antioxidant capacity observed in the German teas compare well with those from other parts of the world such as China, Africa, and Taiwan. This can be inferred from the literature review that we had carried out on our previous investigation regarding European black, green, and white teas and where the same black and green teas (only Korean cultivar) and the white tea had been included [12]. Regarding oolong and yellow teas, there are very few studies on aqueous extracts of these teas, however, the results on TPC are in line with those found by others, despite slightly different extraction methodologies and water/leaf ratios. For comparative purposes, when the data reported in the literature was not in the same units as ours, ours were converted. Zhao et al. found TPC levels in yellow and oolong teas, respectively, of 192 mg GAE/g dry weight (DW) and 108 mg GAE/g DW compared to 289 mg GAE/g DW and 263 mg GAE/g DW found in the yellow and oolong teas of the present study [34]. Chan et al. reported average TPC values on two oolong teas of 8925 mg GAE/100 g tea leaves whereas we found average values of 5254 mg GAE/100 g, bearing in mind that Chan et al. steeped their tea leaves in 80 °C water for 1 h [35]. With regards to the antioxidant capacity using the ABTS assay, Zhao et al. reported values of 1622 μmol TX/g DW and 1210 μmol TX/g DW on yellow and oolong teas, respectively, whereas the values found by us were much higher: 4662 μmol TX/g DW for the yellow tea, and 4177 μmol TX/g DW for the oolong teas [34]. Gramza-Michalowska reported values of 431 mg TX/g DW and 320 mg TX/g DW for yellow and oolong teas, respectively, compared to 234 TX/g DW and 208 TX/g DW for the yellow and oolong German teas [36].

With regards to the three different cultivars (each from three different *C. sinensis* varieties) used for making black and oolong teas, differences in TPC, TFC, and antioxidant capacity were observed. Since the plants are all grown on the same terroir, exposed to the same climatic conditions, and the leaves were harvested and processed in the same way at the same time, the differences must mainly arise from their different genetic background. The *assamica* variety (Azores cultivar) appears to be the most appropriate one in terms of potential health benefits compared to the *sinensis* and *pubilimba* ones as it displayed higher levels of TPC, TFC, and antioxidant activity. This is in accordance with a study by Jin et al. who studied the total catechin content in dried tea leaves of 371 accessions of representative tea germplasms collected in tea-growing provinces of China, and of the 3 varieties, *assamica* had a significantly higher catechin index, a measure of the difference in catechin composition, than the other two, and a higher total catechin content than the *sinensis* variety [2]. In another study examining 107 cultivars belonging to the same three varieties, *assamica* was also shown to be quite different from the *sinensis* and *pubilimba* ones in terms of composition and content of catechins and flavonol glycosides [37]. Other studies have also reported that, in general, the biochemical composition of tea leaves in terms of total catechin and polyphenol content is higher in cultivars from the *assamica* variety than those from the *sinensis* one, reflecting botanical/genetic variability [38,39,40]. The differences in the cultivars observed were partly confirmed by the PCA statistical analysis performed on the tea brews, where the Korean (var. *sinensis*) and Vietnam cultivar (var. *pubilimiba*) were well separated; concerning the Azores cultivar (var. *assamica*) it seems that the type of treatment of the leaves influences the properties of the tea brews more than the cultivar. A separate analysis of the different type of tea can also differentiate these varieties as shown by the pairwise PCA of dry tea leaves of the black and oolong teas performed on ATR-FTIR data that well discriminate the Korean cultivar (var. *sinensis*) from the Azores one (var. *assamica*). This result proves that the different tea varieties have a different chemical fingerprint that can be sufficiently and qualitatively detected by FTIR-ATR spectroscopy, and that these variations can be identified using multivariate analysis with PCA. This is in line with other studies that showed the potential of FTIR spectroscopy to discriminate between different tea varieties [41,42]. 

Two different brewing conditions were used in this study, considering the average household preparation of a cup of hot tea as well as the preparation of cold tea which is becoming increasingly popular as a refreshing cold or iced beverage. Cold brews have a distinct taste and flavour profile, but they still deliver a certain level of beneficial polyphenols but with less bitterness and lower caffeine and tannins [43]. We did not find remarkable significant differences between the two brewing methods except for some exceptions. In general, hot brewing of black tea (var. *assamica*, Azores cultivar) always lead to higher extraction of TPC, TFC, and antioxidant capacity than the other two varieties, indicating that this brewing method would be the one of choice to maximise its health benefits. The opposite is true for white tea where the cold brew always showed to be the more efficient preparation method in terms of health-promoting benefits. These outcomes are in line with our previous studies [44,45]. The reasons underlying these differences, could be due to the fact that during cold brewing, the tearubigins and teaflavins present in fermented and partially fermented teas are likely more difficult to extract since they are high molecular weight polymerization products of catechins, therefore, they could be more easily extracted with hot brewing. However, for non-fermented or slightly fermented teas like white, green tea, and yellow teas, the low molecular weight water-soluble catechins can be easily extracted with cold brewing. Furthermore, in Figure 3 one can observe that cold brewed ORAC activity of green tea is higher than the hot brewed one despite their similar TPC (Figure 2). This peculiarity can be explained by the fact that the ORAC assay measures mostly the HAT capability of compounds which neutralize the peroxyl radicals generated by AAPH. The HAT mechanism can be carried out by polyphenols but not exclusively by them. Hence, other compounds not measured using the Folin–Ciocalteau assay could contribute to antioxidant capacity, especially in the cold brew, where low molecular weight compounds are more easily extractable in the cold brew than high molecular weight ones.

With the rise in tea consumption across the globe, the potential presence of chemical contaminants such as trace elements may raise health concerns [46]. Monitoring the content of PTEs, particularly aluminium, is crucial due to its potential association with the development of Alzheimer’s disease. This is of particular concern because tea has been identified as a hyperaccumulator of this element [47]. Implementing sound cultivation and handling practices for tea leaves can effectively regulate the levels of these contaminants. Specifically, emphasizing the moderate use of pesticides and strategically situating plantations away from anthropogenic sources of contamination can greatly contribute to this endeavor. 

Bearing this in mind, an elemental analysis was also carried out to determine the presence of 15 elements (both potentially toxic and essential) on the German teas. Interestingly, from the PCA analysis, a clear discrimination was observed for the three tea varieties with different accumulations of elements. Previous studies have reported that the geographical origin of tea could be authenticated based on the mineral multi-elements that are translocated from soil to leaves with a significant correlation [48]. However, the German teas studied were all grown on the same soil, hence the results obtained seem to indicate that the different varieties differ in their uptake and concentration of certain elements, depending on the plants homeostasis which is tightly regulated in plant tissues [49]. No discriminatory differences were however noted regarding the degree of oxidation, and this would be expected since the trace elements present in tea leaves cannot be lost during their processing, although they could be transferred in the aqueous phase during brewing. The results of the exposure risk assessment (HQ and HI indexes) showed, however, that there is no carcinogenic risk from consumption of these German teas, hence they would not give rise to health concerns, which in line with our previous investigation on European teas [15]. In fact, it is worth bearing in mind that these indices are calculated on dry leaves which are not consumed, and not on the infusions which contain lower contents of elements since the entire quantity of elements are never fully released in the infusion. 

## 5. Conclusions

Overall, this multi-faceted approach for the characterization of black, green, white, yellow, and oolong teas from three different *Camellia sinensis* varieties grown in Tschanara Tea Garden (Germany) can be a valuable addition to the more commonly known teas grown outside of the European territory, making European teas a promising and attractive option for tea lovers. The results obtained could act as a guide for selecting teas with marked beneficial effects on human health and provide a benchmark for other European tea growers for maintaining standards and targets in the highly competitive global tea market. Furthermore, additional information of the best brewing conditions for maximizing the health benefits of these German teas are provided. Finally, having tea gardens in Europe would help Europeans to visit and learn more about the ancient tea culture and the complex process of producing tea without the need to travel long distances, bridging a gap of knowledge to both tea connoisseurs and non-connoisseurs.

## Figures and Tables

**Table 1:** List of *Camellia sinensis* teas. Type, variety, cultivar, and labeling of the teas studied.

Type	Variety	Cultivar	Label
White	*pubilimba*	Vietnam	WV
Yellow	*sinensis*	Korea	YK
Green	*sinensis*	Korea	GK
Oolong	*assamica*	Azores	OA
	*sinensis*	Korea	OK
Black	*assamica*	Azores	BA
	*pubilimba*	Vietnam	BV
	*sinensis*	Korea	BK

**Table 2:** Total phenolic content (TPC), total flavonoid content (TFC), and antioxidant activity data (ORAC, ABTS, FRAP) of the studied tea brews. Samples are grouped by type of brew and type of tea and the means are reported in bold. Letters within each column indicate homogeneous subclasses resulting from Tukey’s post hoc multiple comparison test (*p* < 0.05) performed between all samples.

Type	Code	TPC (mM GAEq)	TFC (mM CAEq)	ORAC (mM TXEq)	ABTS (mM TXEq)	FRAP (mM AAEq)
	***Cold***	***5.4* ± *2.46***	***0.87* ± *0.413***	***21.8* ± *8.0***	***15.1* ± *5.8***	***10.4* ± *5.23***
black	BA	3.6 ± 0.15 E	0.56 ± 0.028 H	14.6 ± 2.0 I	10.8 ± 1.5 D	6.6 ± 0.87 H
	BV	2.1 ± 0.20 F	0.32 ± 0.025 I	11.0 ± 0.8 J	7.2 ± 0.9 F	4.3 ± 0.72 I
	BK	2.4 ± 0.14 F	0.36 ± 0.020 I	13.3 ± 0.6 I	7.7 ± 1.3 EF	3.5 ± 0.59 I
	**All**	**2.7 ± 0.80**	**0.41 ± 0.131**	**13.0 ± 1.8**	**8.6 ± 2.0**	**4.8 ± 1.59**
green	GK	8.9 ± 0.35 A	1.42 ± 0.115 A	33.2 ± 2.5 A	21.8 ± 3.1 A	17.5 ± 1.78 A
yellow	YK	6.9 ± 0.33 BC	1.06 ± 0.088 D	25.8 ± 1.8 DE	18.7 ± 1.7 B	13.4 ± 1.56 C
oolong	OK	5.4 ± 0.23 D	0.87 ± 0.066 EF	22.5 ± 0.9 FG	15.6 ± 1.8 C	9.6 ± 1.43 F
	OA	6.8 ± 0.56 BC	1.25 ± 0.114 B	27.9 ± 2.2 C	18.7 ± 2.5 B	12.9 ± 1.83 CD
	**All**	**6.1 ± 1.44**	**1.06 ± 0.240**	**25.2 ± 4.5**	**17.1 ± 2.5**	**11.2 ± 3.26**
white	WV	7.1 ± 0.36 B	1.10 ± 0.046 CD	26.5 ± 1.8 CD	20.6 ± 2.2 A	15.4 ± 2.01 B
	***Hot***	***5.8* ± *2.06***	***0.87* ± *0.309***	***20.9* ± *6.5***	***15.2* ± *5.0***	***9.2* ± *3.84***
black	BA	5.5 ± 0.36 D	0.79 ± 0.052 G	16.9 ± 1.1 H	14.0 ± 1.1 C	7.6 ± 0.53 GH
	BV	2.5 ± 0.21 F	0.38 ± 0.028 I	10.5 ± 0.5 J	7.6 ± 0.4 F	4.2 ± 0.23 I
	BK	3.7 ± 0.16 E	0.52 ± 0.036 H	14.7 ± 1.1 I	9.2 ± 0.7 DE	4.4 ± 0.35 I
	**All**	**3.9 ± 1.54**	**0.56 ± 0.211**	**14.1 ± 3.2**	**10.3 ± 3.3**	**5.4 ± 1.92**
green	GK	9.0 ± 0.66 A	1.30 ± 0.093 B	30.4 ± 1.7 B	22.2 ± 2.7 A	15.2 ± 1.14 B
yellow	YK	6.7 ± 0.39 C	1.03 ± 0.075 D	24.3 ± 1.4 EF	18.6 ± 0.8 B	11.8 ± 0.86 DE
oolong	OK	5.3 ± 0.29 D	0.84 ± 0.071 FG	21.8 ± 0.8 G	14.0 ± 1.0 C	8.2 ± 0.53 G
	OA	7.2 ± 0.49 B	1.16 ± 0.074 C	26.6 ± 1.5 CD	18.6 ± 1.0 B	11.0 ± 0.79 E
	**All**	**6.3 ± 1.44**	**1.00 ± 0.180**	**24.2 ± 3.5**	**16.3 ± 3.0**	**9.6 ± 2.65**
white	WV	6.7 ± 0.53 C	0.92 ± 0.058 E	21.7 ± 0.9 G	17.6 ± 1.2 B	11.5 ± 0.98 E

**Table 3:** Matrix of Pearson’s correlation coefficients (a) and (b) relative *p*-values (values are all different from 0 with a significance level alpha = 0.05).

**(a)**	**Variables**	**TPC**	**TFC**	**ORAC**	**ABTS**	**FRAP**
	TPC	1	0.981	0.966	0.987	0.956
	TFC	0.981	1	0.986	0.977	0.955
	ORAC	0.966	0.986	1	0.967	0.957
	ABTS	0.987	0.977	0.967	1	0.976
	FRAP	0.956	0.955	0.957	0.976	1
**(b)**	**Variables**	**TPC**	**TFC**	**ORAC**	**ABTS**	**FRAP**
	TPC	0	<0.0001	<0.0001	<0.0001	<0.0001
	TFC	<0.0001	0	<0.0001	<0.0001	<0.0001
	ORAC	<0.0001	<0.0001	0	<0.0001	<0.0001
	ABTS	<0.0001	<0.0001	<0.0001	0	<0.0001
	FRAP	<0.0001	<0.0001	<0.0001	<0.0001	0

**Table 4:** List of the most significant IR peaks together with the corresponding vibrational modes and their biological attribution.

Peak Position (cm^−1^)	Vibrational Mode	Biological Attribution
~2921, and ~2849	ν_asym_ and ν_sym_ CH_2_	Lipids
~1739	ν C=O ester	Pheophytin
~1703	ν C=O	Chlorophyll
~1623 (~1644, ~1624, and ~1603)	ν C=O, ν C-N and δ N-H (Amide I)	CONH groups in alkaloids
~1514	ν conjugated C=C	Polyphenols
~1237	phenyl ring breathing vibration	
~1451, ~1368, and ~1315	δ C-H	Cellulose
~1033	ν C-OH	
~1147	ν O-C-O	Carbohydrates

## Data Availability

The data presented in this study are available in the Supplementary Materials.

## Supplementary Materials

The following supporting information can be downloaded at: https://www.mdpi.com/article/10.3390/antiox12111943/s1, Figure S1: Averaged UV-Vis spectra (200–500 nm, Δλ = 2 nm) of the cold tea brews (cultivar: Korea); Figure S2: Correlogram plot on element content in Tschanara Tea Garden tea leaves; Table S1: Mineral composition of the mineral water “Sant’Anna”; Table S2: UV-Vis absorption spectra of the hot and cold brews (200–500 nm); Table S3: Eigenvalues and explained and cumulative variance, for the first five principal components (PC) obtained from the analysis of the total phenol (TPC) and flavonoid (TFC) content, antioxidant activity (ORAC, ABTS, FRAP), and UV-Vis absorptions (200–500 nm) of the three cold brews obtained from each of the eight tea samples; Table S4: Essential elements content (mg kg^−1^) in differently processed tea leaves from Tschanara Tea Garden; Table S5: Potentially toxic elements content (mg kg^−1^) in differently processed tea leaves from Tschanara Tea Garden; Table S6: Principal component analysis. Eigenvalues, explained and cumulative variance; Table S7: Estimated hazard quotient (HQ) and hazard index (HI) of exposure to elements from consumption of Tschanara Tea Garden teas by European consumers.
