# Supplementary material for: Insights on the Nutraceutical Properties of Different Specialty Teas Grown and Processed in a German Tea Garden
Source: Antioxidants (Basel). 2023 Oct 31;12(11):1943. doi: 10.3390/antiox12111943 (PMC10669839; doi:10.3390/antiox12111943)
Supplement: Supplementary file 1 [file antioxidants-12-01943-s001.zip › antioxidants-2659542-supplementary.pdf]

Figure S1. Averaged UV-Vis spectra (200–500 nm, Δλ = 2 nm) of the cold tea brews (Cultivar: Korea). Spectra are colored according to the type of tea (brown = black tea; green = green tea; yellow = yellow tea; rusty = oolong tea). In the zoomed and spaced insert, absorptions from 300 to 440 nm are reported.

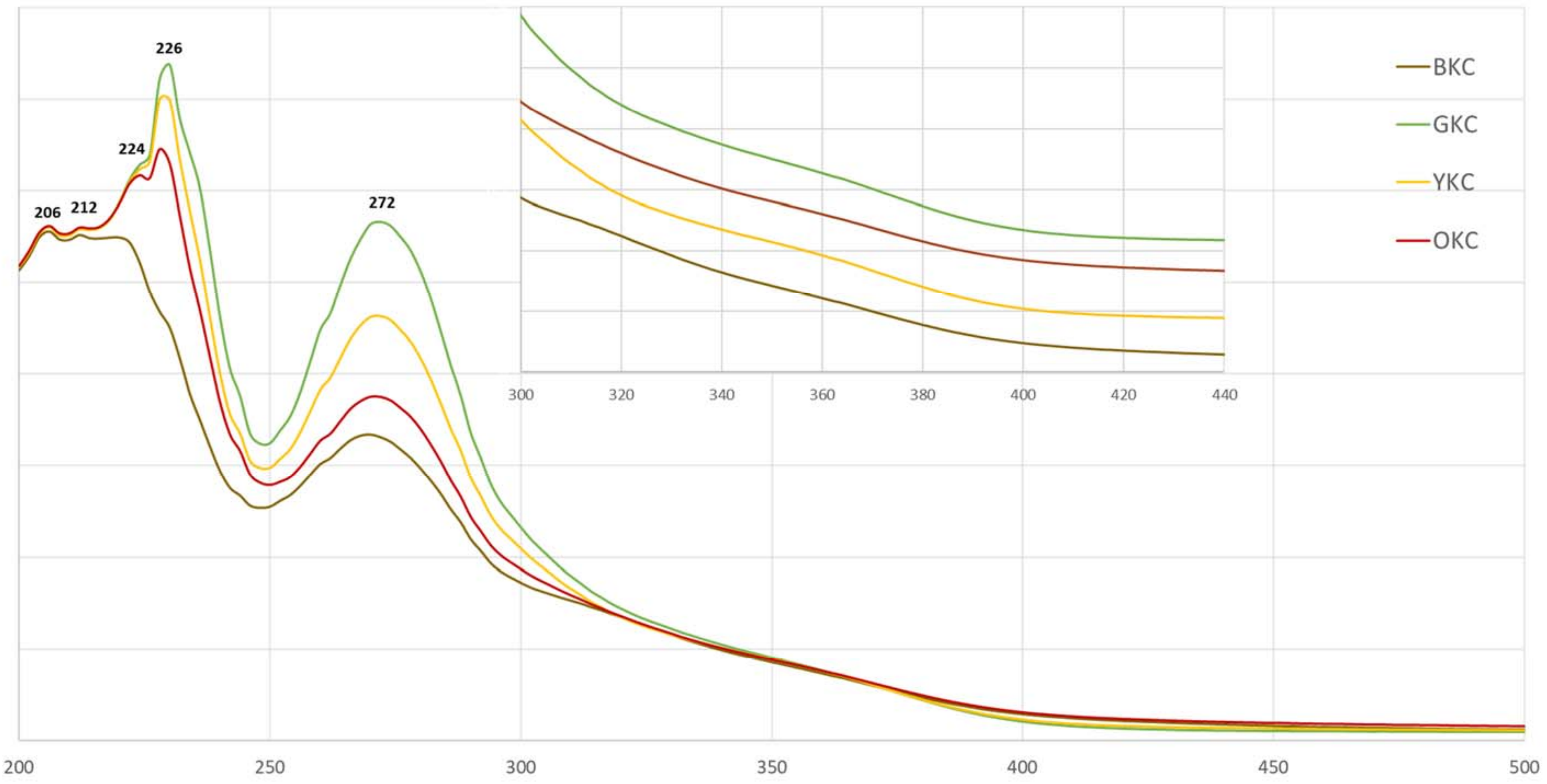

Figure S2. Correlogram plot on element content in Tschanara Tea Garden tea leaves. * denotes a statistically significant correlation at 95% confidence level. ** denotes a statistically significant correlation at 99% confidence level.

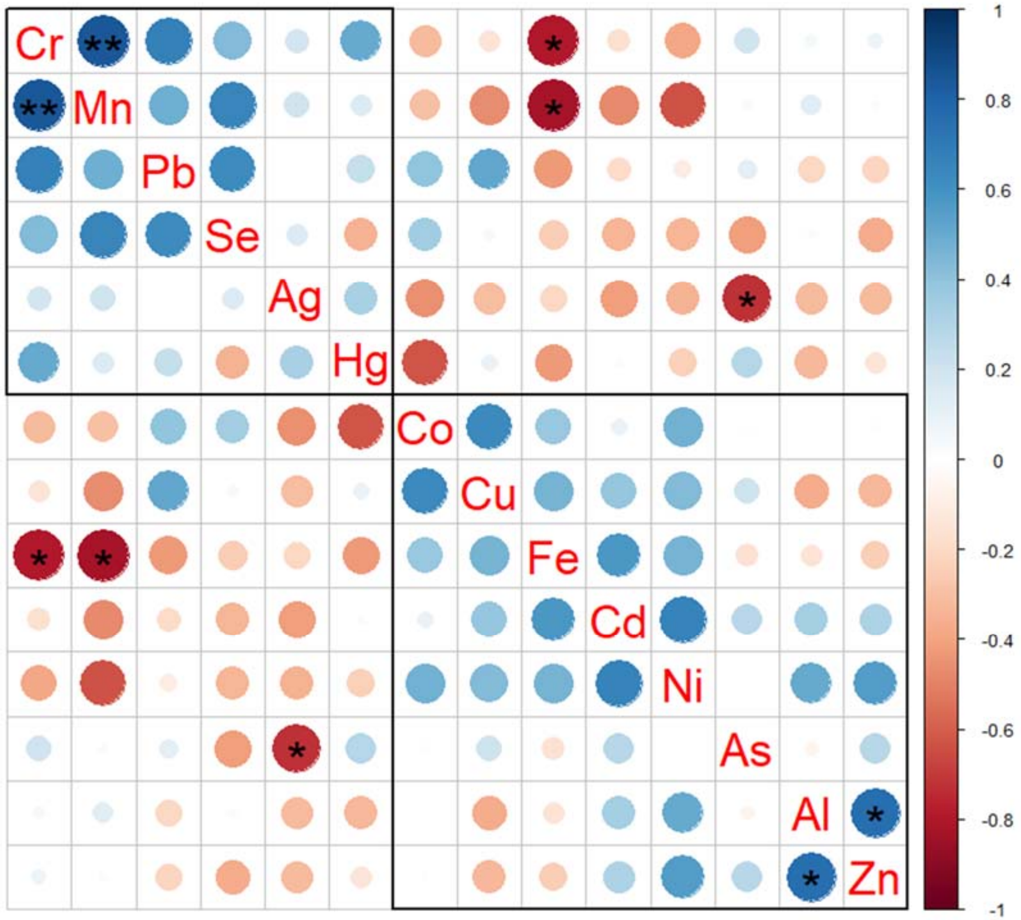

Table S1: Water composition "Sant'Anna"

pH	6.8
Total hardness	0.98 F
Electric conductivity	25.5 µS/cm
Free CO_2	3.0 mg/L
Fixed residue at 180 °C	22 mg/L
Bicarbonates	11 mg/L
Calcium	3.3 mg/L
Nitrates	0.88 mg/L
Sodium	1.5 mg/L
Fluorides	<0.10 mg/L
Ammonium and Nitrites	not detected

Label on Bottle with water composition and other parameters

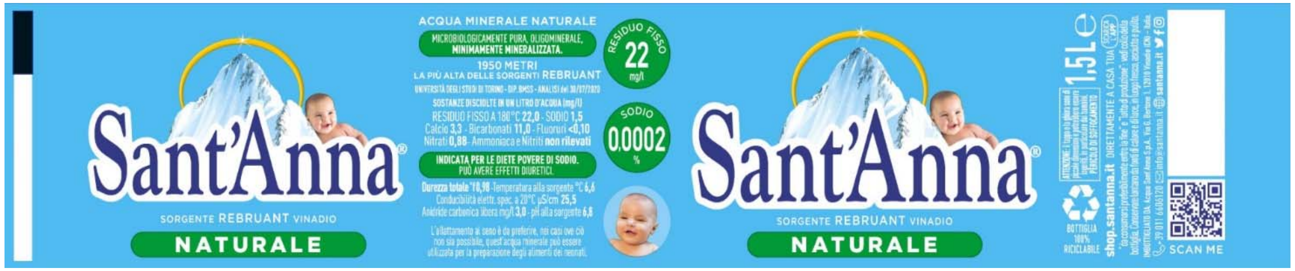

Table S2. UV-VIS absorption spectra of the hot and cold brews (200-500 nm) recorded in duplicate at constant intervals (Δλ = 2 nm) against water as blank on the infusions prepared independently three times and reported as mean values.

nm	BAH	BVH	BKH	GKH	YKH	OKH	OAH	WVH	BAC	BVC	BKC	GKC	YKC	OKC	OAC	WVC
200	2.607	2.542	2.606	2.621	2.598	2.618	2.586	2.548	2.554	2.516	2.570	2.582	2.586	2.592	2.582	2.548
202	2.689	2.624	2.688	2.709	2.678	2.703	2.673	2.635	2.634	2.592	2.648	2.667	2.664	2.675	2.665	2.635
204	2.799	2.730	2.793	2.802	2.780	2.807	2.776	2.737	2.738	2.695	2.750	2.767	2.767	2.775	2.768	2.735
206	2.832	2.761	2.830	2.847	2.814	2.843	2.814	2.773	2.771	2.727	2.781	2.804	2.802	2.809	2.809	2.773
208	2.787	2.715	2.788	2.801	2.783	2.800	2.771	2.732	2.729	2.683	2.737	2.758	2.759	2.769	2.763	2.728
210	2.788	2.711	2.784	2.807	2.782	2.806	2.776	2.732	2.722	2.677	2.735	2.761	2.758	2.769	2.760	2.731
212	2.819	2.741	2.814	2.833	2.805	2.831	2.802	2.759	2.751	2.700	2.762	2.793	2.792	2.800	2.797	2.760
214	2.825	2.728	2.812	2.837	2.799	2.826	2.801	2.755	2.747	2.686	2.744	2.791	2.787	2.796	2.796	2.754
216	2.830	2.727	2.820	2.845	2.817	2.838	2.809	2.764	2.759	2.677	2.742	2.804	2.798	2.804	2.803	2.764
218	2.875	2.750	2.861	2.891	2.859	2.883	2.855	2.808	2.798	2.678	2.747	2.845	2.841	2.849	2.855	2.808
220	2.971	2.769	2.941	2.983	2.951	2.970	2.947	2.898	2.868	2.656	2.747	2.940	2.936	2.932	2.944	2.899
222	3.092	2.761	3.029	3.107	3.067	3.091	3.064	3.008	2.942	2.593	2.720	3.061	3.047	3.039	3.062	3.013
224	3.162	2.648	3.042	3.190	3.134	3.154	3.129	3.063	2.927	2.434	2.609	3.142	3.116	3.084	3.143	3.086
226	3.216	2.464	2.985	3.255	3.177	3.187	3.165	3.073	2.824	2.228	2.451	3.194	3.158	3.068	3.201	3.121
228	3.549	2.328	3.014	3.668	3.518	3.450	3.468	3.283	2.788	2.075	2.340	3.614	3.501	3.226	3.568	3.418
230	3.533	2.226	2.929	3.752	3.502	3.412	3.489	3.197	2.675	1.977	2.252	3.690	3.493	3.146	3.580	3.380
232	3.212	2.032	2.691	3.446	3.178	3.106	3.190	2.830	2.426	1.805	2.085	3.394	3.167	2.864	3.278	3.027
234	2.948	1.813	2.445	3.264	2.876	2.818	2.947	2.476	2.158	1.612	1.893	3.208	2.890	2.577	3.041	2.698
236	2.715	1.658	2.262	3.066	2.607	2.582	2.731	2.208	1.957	1.476	1.750	3.013	2.623	2.348	2.817	2.436
238	2.456	1.505	2.071	2.749	2.291	2.314	2.456	1.937	1.752	1.345	1.606	2.677	2.308	2.092	2.523	2.153
240	2.212	1.366	1.896	2.409	1.993	2.055	2.188	1.694	1.561	1.226	1.472	2.313	2.003	1.848	2.231	1.897
242	2.042	1.273	1.778	2.142	1.780	1.868	1.992	1.532	1.427	1.146	1.378	2.028	1.782	1.671	2.009	1.726
244	1.957	1.236	1.723	1.996	1.673	1.776	1.891	1.458	1.368	1.113	1.336	1.882	1.676	1.581	1.893	1.650
246	1.836	1.190	1.648	1.805	1.538	1.639	1.742	1.375	1.283	1.079	1.281	1.675	1.528	1.452	1.716	1.560
248	1.806	1.189	1.633	1.752	1.497	1.597	1.699	1.358	1.262	1.077	1.268	1.621	1.487	1.407	1.661	1.547
250	1.813	1.208	1.643	1.755	1.498	1.591	1.697	1.374	1.266	1.094	1.275	1.624	1.489	1.394	1.650	1.569
252	1.863	1.263	1.688	1.835	1.555	1.619	1.739	1.449	1.297	1.145	1.308	1.693	1.537	1.412	1.676	1.650
254	1.919	1.315	1.731	1.917	1.610	1.658	1.791	1.516	1.333	1.188	1.339	1.769	1.591	1.438	1.718	1.721
256	2.015	1.393	1.802	2.061	1.711	1.731	1.884	1.629	1.395	1.256	1.389	1.901	1.683	1.493	1.800	1.833
258	2.139	1.489	1.890	2.247	1.839	1.825	2.006	1.772	1.472	1.339	1.450	2.068	1.798	1.565	1.908	1.971
260	2.272	1.585	1.979	2.442	1.969	1.923	2.134	1.919	1.549	1.418	1.509	2.240	1.914	1.637	2.022	2.108
262	2.342	1.640	2.026	2.540	2.035	1.977	2.204	1.994	1.594	1.461	1.542	2.331	1.978	1.675	2.086	2.176

264	2.458	1.724	2.102	2.708	2.155	2.068	2.322	2.122	1.667	1.530	1.594	2.485	2.085	1.744	2.195	2.286
266	2.571	1.807	2.169	2.857	2.274	2.155	2.428	2.255	1.741	1.599	1.640	2.629	2.189	1.811	2.307	2.388
268	2.659	1.867	2.213	2.981	2.361	2.218	2.515	2.356	1.793	1.644	1.665	2.736	2.264	1.853	2.391	2.465
270	2.726	1.906	2.233	3.096	2.422	2.260	2.584	2.432	1.830	1.672	1.671	2.818	2.310	1.877	2.456	2.513
272	2.753	1.921	2.218	3.109	2.444	2.270	2.606	2.472	1.846	1.680	1.657	2.832	2.315	1.877	2.488	2.529
274	2.747	1.911	2.188	3.094	2.429	2.254	2.598	2.476	1.843	1.668	1.632	2.810	2.293	1.857	2.488	2.513
276	2.695	1.866	2.129	3.038	2.379	2.207	2.547	2.437	1.816	1.628	1.589	2.750	2.238	1.816	2.454	2.459
278	2.634	1.804	2.062	2.960	2.317	2.147	2.486	2.378	1.776	1.574	1.543	2.676	2.174	1.768	2.405	2.388
280	2.526	1.711	1.973	2.850	2.221	2.061	2.388	2.279	1.712	1.496	1.485	2.561	2.083	1.700	2.318	2.284
282	2.406	1.601	1.874	2.710	2.102	1.955	2.271	2.159	1.633	1.404	1.419	2.413	1.970	1.617	2.207	2.158
284	2.249	1.477	1.762	2.509	1.960	1.833	2.115	2.009	1.540	1.300	1.346	2.234	1.842	1.525	2.067	2.008
286	2.071	1.331	1.635	2.302	1.807	1.696	1.948	1.843	1.433	1.179	1.262	2.047	1.702	1.421	1.910	1.840
288	1.914	1.204	1.522	2.114	1.670	1.578	1.796	1.690	1.339	1.072	1.189	1.882	1.583	1.330	1.769	1.691
290	1.719	1.048	1.384	1.887	1.507	1.431	1.609	1.505	1.223	0.942	1.097	1.679	1.434	1.219	1.593	1.505
292	1.583	0.941	1.288	1.729	1.390	1.329	1.480	1.371	1.141	0.852	1.034	1.542	1.332	1.141	1.472	1.374
294	1.438	0.828	1.187	1.563	1.270	1.224	1.341	1.227	1.055	0.759	0.966	1.398	1.227	1.064	1.349	1.232
296	1.336	0.751	1.116	1.445	1.184	1.148	1.245	1.120	0.997	0.696	0.921	1.299	1.153	1.010	1.264	1.126
298	1.262	0.700	1.067	1.356	1.117	1.094	1.175	1.037	0.955	0.651	0.889	1.229	1.099	0.971	1.203	1.044
300	1.193	0.655	1.023	1.272	1.056	1.043	1.108	0.957	0.915	0.613	0.861	1.161	1.047	0.935	1.145	0.963
302	1.128	0.616	0.983	1.191	0.995	0.994	1.045	0.877	0.878	0.580	0.834	1.096	0.995	0.900	1.091	0.884
304	1.075	0.585	0.952	1.123	0.942	0.952	0.991	0.808	0.849	0.554	0.815	1.042	0.951	0.872	1.048	0.815
306	1.025	0.557	0.924	1.056	0.890	0.913	0.940	0.741	0.823	0.531	0.797	0.991	0.909	0.845	1.007	0.749
308	0.974	0.529	0.895	0.989	0.837	0.872	0.888	0.671	0.796	0.507	0.779	0.939	0.865	0.818	0.965	0.683
310	0.930	0.506	0.871	0.932	0.792	0.838	0.844	0.613	0.772	0.487	0.763	0.896	0.828	0.793	0.928	0.629
312	0.889	0.485	0.848	0.881	0.749	0.806	0.803	0.560	0.747	0.468	0.748	0.856	0.794	0.770	0.893	0.580
314	0.843	0.460	0.821	0.829	0.708	0.771	0.759	0.507	0.718	0.446	0.728	0.814	0.757	0.745	0.854	0.530
316	0.806	0.442	0.800	0.789	0.674	0.744	0.725	0.466	0.693	0.429	0.712	0.781	0.728	0.723	0.822	0.492
318	0.766	0.421	0.776	0.750	0.642	0.716	0.689	0.428	0.663	0.410	0.692	0.747	0.698	0.700	0.787	0.455
320	0.733	0.405	0.755	0.718	0.616	0.693	0.661	0.398	0.637	0.394	0.674	0.720	0.675	0.679	0.758	0.427
322	0.698	0.388	0.733	0.690	0.593	0.669	0.632	0.372	0.607	0.377	0.653	0.693	0.650	0.658	0.726	0.401
324	0.667	0.374	0.713	0.666	0.572	0.649	0.608	0.351	0.579	0.362	0.634	0.670	0.630	0.638	0.697	0.380
326	0.638	0.361	0.694	0.645	0.555	0.631	0.586	0.334	0.551	0.347	0.615	0.649	0.612	0.620	0.668	0.362
328	0.612	0.349	0.677	0.627	0.540	0.614	0.566	0.320	0.525	0.334	0.596	0.630	0.595	0.602	0.641	0.347
330	0.585	0.337	0.658	0.608	0.526	0.597	0.545	0.308	0.496	0.321	0.577	0.610	0.579	0.584	0.611	0.332
332	0.558	0.325	0.640	0.590	0.512	0.580	0.525	0.297	0.467	0.307	0.558	0.590	0.562	0.566	0.581	0.318

334	0.535	0.316	0.625	0.574	0.499	0.566	0.507	0.288	0.442	0.295	0.541	0.573	0.548	0.550	0.553	0.306
336	0.511	0.306	0.609	0.557	0.487	0.551	0.490	0.279	0.416	0.284	0.524	0.555	0.533	0.534	0.525	0.294
338	0.489	0.297	0.595	0.541	0.475	0.537	0.472	0.271	0.391	0.273	0.508	0.539	0.520	0.519	0.497	0.283
340	0.468	0.289	0.580	0.525	0.463	0.523	0.456	0.263	0.368	0.262	0.493	0.523	0.506	0.505	0.470	0.272
342	0.449	0.280	0.567	0.510	0.452	0.510	0.440	0.256	0.347	0.253	0.478	0.508	0.493	0.491	0.444	0.262
344	0.430	0.272	0.553	0.495	0.441	0.497	0.424	0.249	0.326	0.243	0.464	0.492	0.480	0.478	0.419	0.252
346	0.414	0.266	0.541	0.481	0.430	0.485	0.410	0.241	0.308	0.235	0.452	0.479	0.469	0.465	0.396	0.243
348	0.398	0.259	0.530	0.467	0.419	0.474	0.396	0.235	0.292	0.228	0.440	0.465	0.457	0.453	0.375	0.235
350	0.383	0.253	0.516	0.453	0.409	0.461	0.382	0.229	0.275	0.220	0.427	0.451	0.445	0.441	0.354	0.227
352	0.369	0.247	0.504	0.440	0.398	0.450	0.370	0.223	0.262	0.213	0.414	0.438	0.432	0.429	0.335	0.219
354	0.356	0.241	0.491	0.426	0.387	0.438	0.357	0.217	0.248	0.206	0.402	0.423	0.420	0.417	0.316	0.211
356	0.344	0.235	0.478	0.414	0.376	0.426	0.345	0.212	0.236	0.200	0.390	0.410	0.407	0.405	0.300	0.205
358	0.333	0.230	0.464	0.401	0.364	0.414	0.334	0.206	0.226	0.194	0.377	0.396	0.393	0.393	0.285	0.198
360	0.322	0.225	0.451	0.388	0.353	0.401	0.323	0.201	0.216	0.189	0.365	0.381	0.379	0.380	0.271	0.191
362	0.312	0.220	0.436	0.375	0.341	0.389	0.311	0.196	0.206	0.184	0.352	0.366	0.365	0.367	0.258	0.185
364	0.305	0.217	0.424	0.363	0.329	0.378	0.302	0.191	0.200	0.179	0.340	0.353	0.352	0.355	0.248	0.180
366	0.295	0.212	0.408	0.350	0.317	0.365	0.291	0.187	0.192	0.174	0.327	0.337	0.336	0.341	0.236	0.173
368	0.286	0.207	0.392	0.337	0.304	0.352	0.281	0.182	0.185	0.170	0.313	0.321	0.320	0.327	0.225	0.167
370	0.278	0.202	0.376	0.324	0.291	0.339	0.270	0.176	0.177	0.165	0.299	0.303	0.303	0.313	0.215	0.161
372	0.269	0.198	0.360	0.311	0.278	0.326	0.260	0.172	0.171	0.160	0.285	0.286	0.287	0.299	0.206	0.155
374	0.262	0.193	0.345	0.298	0.265	0.314	0.250	0.167	0.165	0.156	0.271	0.269	0.271	0.285	0.197	0.150
376	0.253	0.189	0.329	0.284	0.252	0.301	0.240	0.162	0.159	0.152	0.258	0.252	0.254	0.272	0.188	0.144
378	0.246	0.185	0.314	0.272	0.240	0.289	0.231	0.157	0.154	0.147	0.245	0.236	0.239	0.258	0.180	0.139
380	0.240	0.181	0.299	0.259	0.227	0.278	0.222	0.153	0.149	0.144	0.233	0.220	0.224	0.246	0.173	0.134
382	0.233	0.177	0.284	0.247	0.216	0.266	0.213	0.148	0.144	0.140	0.220	0.204	0.208	0.233	0.166	0.129
384	0.226	0.172	0.270	0.235	0.205	0.255	0.204	0.144	0.139	0.136	0.209	0.188	0.194	0.222	0.159	0.123
386	0.220	0.169	0.257	0.224	0.194	0.245	0.196	0.139	0.135	0.132	0.198	0.174	0.181	0.210	0.152	0.118
388	0.213	0.165	0.245	0.213	0.184	0.235	0.188	0.135	0.131	0.129	0.188	0.161	0.169	0.200	0.146	0.114
390	0.208	0.162	0.234	0.204	0.176	0.226	0.182	0.131	0.128	0.126	0.179	0.149	0.157	0.191	0.141	0.110
392	0.202	0.158	0.223	0.195	0.167	0.217	0.174	0.127	0.124	0.123	0.169	0.137	0.146	0.182	0.136	0.105
394	0.197	0.155	0.213	0.186	0.159	0.210	0.168	0.124	0.120	0.120	0.162	0.127	0.137	0.174	0.131	0.102
396	0.192	0.152	0.204	0.179	0.152	0.203	0.162	0.120	0.117	0.117	0.155	0.118	0.128	0.166	0.126	0.098
398	0.187	0.149	0.196	0.172	0.146	0.196	0.157	0.118	0.115	0.115	0.148	0.109	0.121	0.160	0.122	0.095
400	0.182	0.146	0.189	0.166	0.141	0.191	0.152	0.115	0.112	0.112	0.142	0.102	0.115	0.154	0.118	0.092
402	0.177	0.143	0.181	0.160	0.136	0.185	0.146	0.112	0.109	0.110	0.137	0.095	0.108	0.148	0.114	0.089

404	0.173	0.140	0.175	0.155	0.132	0.180	0.142	0.109	0.106	0.108	0.132	0.090	0.103	0.143	0.111	0.087
406	0.169	0.138	0.169	0.151	0.128	0.176	0.138	0.107	0.104	0.106	0.128	0.085	0.099	0.139	0.108	0.084
408	0.166	0.136	0.164	0.147	0.125	0.172	0.135	0.105	0.103	0.104	0.124	0.081	0.095	0.135	0.106	0.083
410	0.162	0.132	0.159	0.143	0.122	0.168	0.131	0.102	0.100	0.101	0.120	0.077	0.091	0.131	0.103	0.080
412	0.158	0.131	0.155	0.140	0.118	0.165	0.128	0.101	0.098	0.099	0.117	0.074	0.089	0.128	0.101	0.079
414	0.154	0.128	0.150	0.137	0.116	0.161	0.125	0.099	0.096	0.098	0.113	0.071	0.086	0.125	0.099	0.077
416	0.151	0.125	0.146	0.135	0.115	0.158	0.122	0.097	0.094	0.096	0.110	0.068	0.083	0.122	0.096	0.075
418	0.148	0.124	0.143	0.133	0.113	0.156	0.121	0.096	0.093	0.094	0.108	0.066	0.082	0.120	0.095	0.074
420	0.145	0.121	0.139	0.130	0.111	0.153	0.118	0.094	0.091	0.092	0.105	0.064	0.080	0.117	0.093	0.073
422	0.143	0.120	0.136	0.127	0.109	0.151	0.116	0.093	0.090	0.091	0.103	0.062	0.079	0.116	0.091	0.072
424	0.140	0.118	0.133	0.126	0.107	0.149	0.114	0.091	0.088	0.089	0.101	0.061	0.077	0.114	0.089	0.071
426	0.137	0.116	0.130	0.123	0.106	0.147	0.112	0.090	0.087	0.088	0.099	0.060	0.076	0.112	0.088	0.070
428	0.135	0.114	0.127	0.121	0.104	0.144	0.110	0.089	0.085	0.086	0.097	0.058	0.074	0.110	0.087	0.069
430	0.132	0.112	0.124	0.119	0.103	0.142	0.108	0.088	0.083	0.084	0.095	0.057	0.073	0.108	0.085	0.067
432	0.129	0.110	0.121	0.117	0.102	0.140	0.106	0.086	0.082	0.083	0.092	0.056	0.072	0.106	0.083	0.066
434	0.126	0.109	0.119	0.116	0.100	0.138	0.104	0.085	0.081	0.081	0.091	0.055	0.071	0.105	0.082	0.066
436	0.124	0.107	0.116	0.114	0.099	0.136	0.103	0.084	0.079	0.080	0.089	0.055	0.071	0.104	0.081	0.065
438	0.121	0.105	0.114	0.113	0.099	0.134	0.101	0.083	0.078	0.079	0.088	0.053	0.070	0.102	0.080	0.064
440	0.119	0.103	0.111	0.112	0.098	0.133	0.099	0.082	0.076	0.077	0.086	0.053	0.069	0.101	0.078	0.063
442	0.117	0.102	0.109	0.110	0.096	0.131	0.098	0.081	0.076	0.076	0.084	0.053	0.069	0.100	0.078	0.063
444	0.114	0.100	0.106	0.110	0.096	0.129	0.096	0.080	0.074	0.075	0.083	0.052	0.068	0.098	0.076	0.062
446	0.112	0.098	0.104	0.109	0.095	0.128	0.094	0.079	0.073	0.074	0.081	0.052	0.067	0.098	0.075	0.061
448	0.109	0.097	0.102	0.107	0.094	0.126	0.093	0.078	0.072	0.072	0.080	0.051	0.067	0.096	0.074	0.060
450	0.107	0.095	0.100	0.107	0.093	0.125	0.092	0.077	0.071	0.071	0.078	0.051	0.066	0.096	0.073	0.060
452	0.105	0.094	0.098	0.107	0.093	0.124	0.090	0.077	0.070	0.071	0.077	0.051	0.066	0.095	0.072	0.059
454	0.103	0.093	0.096	0.106	0.092	0.123	0.089	0.075	0.069	0.069	0.076	0.050	0.065	0.094	0.071	0.058
456	0.101	0.091	0.094	0.105	0.092	0.121	0.087	0.075	0.068	0.068	0.075	0.050	0.064	0.093	0.070	0.058
458	0.099	0.089	0.092	0.105	0.091	0.120	0.086	0.074	0.066	0.067	0.074	0.049	0.064	0.092	0.069	0.057
460	0.096	0.088	0.090	0.103	0.091	0.118	0.085	0.073	0.065	0.066	0.072	0.049	0.063	0.091	0.068	0.057
462	0.096	0.088	0.089	0.103	0.090	0.117	0.084	0.073	0.065	0.066	0.072	0.049	0.063	0.090	0.068	0.056
464	0.094	0.086	0.088	0.102	0.090	0.116	0.082	0.072	0.064	0.065	0.071	0.049	0.063	0.090	0.067	0.056
466	0.092	0.085	0.086	0.101	0.089	0.115	0.081	0.071	0.063	0.064	0.070	0.048	0.062	0.089	0.066	0.055
468	0.090	0.084	0.084	0.100	0.088	0.114	0.080	0.070	0.062	0.063	0.068	0.048	0.061	0.088	0.065	0.054
470	0.089	0.083	0.083	0.100	0.087	0.113	0.079	0.070	0.061	0.062	0.068	0.048	0.061	0.087	0.064	0.054
472	0.088	0.082	0.082	0.100	0.087	0.112	0.079	0.070	0.061	0.062	0.068	0.048	0.061	0.087	0.064	0.054

474	0.086	0.081	0.080	0.099	0.086	0.110	0.077	0.068	0.060	0.061	0.066	0.047	0.060	0.085	0.062	0.053
476	0.084	0.079	0.079	0.098	0.086	0.110	0.076	0.068	0.059	0.060	0.065	0.047	0.060	0.084	0.062	0.053
478	0.084	0.080	0.079	0.098	0.086	0.109	0.075	0.068	0.059	0.060	0.065	0.047	0.060	0.085	0.062	0.053
480	0.082	0.079	0.078	0.098	0.085	0.108	0.075	0.067	0.058	0.060	0.064	0.047	0.059	0.084	0.061	0.052
482	0.081	0.077	0.077	0.098	0.086	0.107	0.074	0.067	0.058	0.059	0.064	0.047	0.059	0.083	0.060	0.052
484	0.079	0.076	0.075	0.098	0.085	0.106	0.073	0.066	0.057	0.058	0.063	0.046	0.059	0.083	0.059	0.051
486	0.079	0.077	0.074	0.096	0.083	0.105	0.072	0.065	0.057	0.058	0.062	0.047	0.059	0.081	0.059	0.051
488	0.077	0.075	0.073	0.096	0.084	0.104	0.071	0.065	0.056	0.057	0.061	0.046	0.058	0.081	0.058	0.051
490	0.077	0.076	0.073	0.096	0.083	0.104	0.071	0.065	0.056	0.057	0.061	0.046	0.059	0.081	0.058	0.051
492	0.075	0.074	0.072	0.095	0.083	0.103	0.069	0.065	0.055	0.056	0.060	0.046	0.058	0.080	0.057	0.050
494	0.074	0.074	0.071	0.095	0.081	0.102	0.069	0.064	0.055	0.056	0.060	0.046	0.058	0.079	0.057	0.050
496	0.073	0.072	0.070	0.095	0.082	0.101	0.068	0.064	0.054	0.055	0.059	0.046	0.057	0.079	0.056	0.049
498	0.072	0.072	0.069	0.094	0.081	0.100	0.067	0.063	0.054	0.055	0.059	0.046	0.057	0.078	0.056	0.049
500	0.071	0.071	0.069	0.093	0.080	0.099	0.066	0.063	0.053	0.054	0.058	0.045	0.056	0.077	0.055	0.049

Table S3. Eigenvalues and explained and cumulative variance, for the first five principal components (PC) obtained from the analysis of the total phenol (TPC) and flavonoid (TFC) content, antioxidant activity (ORAC, ABTS, FRAP) and UV-Vis absorptions (200-500 nm) of the three cold brews obtained from each of the eight tea samples.

Principal Component Analysis:

	F1	F2	F3	F4	F5
Eigenvalue	72.999	63.561	12.126	4.572	1.412
Variability (%)	46.794	40.744	7.773	2.931	0.905
Cumulative %	46.794	87.538	95.312	98.242	99.147

Table S4. Essential elements content (mg kg^{-1}) in differently processed tea leaves from Tschanara Tea Garden.

	Co	Cu	Fe	Mn	Se	V	Zn
BA	0.05±0.01^{d}	13.3±0.5^{b}	76±9^{a}	375±2^{g}	0.09±0.01^{a}	<0.006	23±1^{d}
BK	0.09±0.002^{c}	12.2±0.3^{bc}	49±1^{de}	628±5^{e}	0.09±0.02^{a}	<0.006	33.2±0.3^{b}
BV	0.25±0.01^{a}	16.9±0.3^{a}	68±2^{ab}	535±9^{f}	0.14±0.02^{a}	<0.006	21.9±0.2^{d}
GK	0.06±0.01^{d}	13±0.3^{b}	50±2^{de}	709±9^{c}	0.11±0.03^{a}	<0.006	24.3±0.1^{c}
OA	0.11±0.01^{c}	11.3±0.6^{cd}	59±3^{bcd}	680±5^{d}	0.118±0.001^{a}	<0.006	38.2±0.2^{a}
OK	0.061±0.002^{d}	11.8±0.3^{cd}	45±2^{e}	1086±2^{a}	0.14±0.05^{a}	<0.006	24.1±0.1^{c}
WV	0.15±0.01^{b}	11±0.1^{d}	61±1^{bc}	684±1^{d}	0.13±0.02^{a}	<0.006	23.9±0.2^{c}
YK	0.05±0.01^{d}	11.7±0.6^{cd}	56±3^{c}	778±4^{b}	0.13±0.02^{a}	<0.006	19.3±0.3^{e}

Table S5. Potentially toxic elements content (mg kg^{-1}) in differently processed tea leaves from Tschanara Tea Garden.

	Ag	Al	As	Cd	Cr	Hg	Ni	Pb
BA	0.0066±0.0003^{b}	1680±158^{e}	0.1±0.01^{a}	0.071±0.004^{a}	0.341±0.005^{d}	0.01±0.001^{b}	10.2±0.2^{b}	0.24±0.02^{e}
BK	0.0068±0.0002^{b}	2167±83d^{e}	0.1±0.02^{a}	0.032±0.001^{d}	0.47±0.01^{b}	0.012±0.002^{ab}	10±0.3^{b}	0.42±0.03^{cd}
BV	0.0064±0.0001^{b}	1843±46^{e}	0.09±0.01^{a}	0.054±0.002^{b}	0.43±0.01^{c}	0.0068±0.0003^{c}	12.2±0.1^{a}	0.61±0.04^{a}
GK	0.0068±0.0001^{b}	4865±933^{b}	0.09±0.01^{a}	0.037±0.002^{cd}	0.49±0.03^{b}	0.0145±0.0003^{a}	9.8±0.2^{b}	0.46±0.03^{bc}
OA	0.0066±0.0002^{b}	10582±845^{a}	0.09±0.02^{a}	0.0669±0.0002^{a}	0.46±0.01^{bc}	0.0059±0.0004^{c}	13.4±0.5^{a}	0.36±0.02^{cde}
OK	0.00658±0.00004^{b}	3300±100^{cd}	0.1±0.004^{a}	0.04±0.001^{c}	0.64±0.02^{a}	0.011±0.001^{b}	6.2±0.2^{d}	0.56±0.08^{ab}
WV	0.0065±0.0001^{b}	4171±117^{bc}	0.09±0.03^{a}	0.019±0.001^{f}	0.337±0.005^{d}	0.0021±0.0003^{d}	8±1^{c}	0.31±0.03^{de}
YK	0.0083±0.0003^{a}	955±16^{e}	0.08±0.01^{a}	0.025±0.001^{e}	0.49±0.01^{b}	0.011±0.002^{b}	7.8±0.1^{c}	0.44±0.06^{bc}

Table S6. Principal Component Analysis. Eigenvalues, explained and cumulative variance.

	Principal components			
	1	2	3	4
Variance explained				
Eigenvalues	2.1628	1.7346	1.5664	1.4417
% of variance	33.41	21.49	17.53	14.85
Cumulative %	33.41	54.90	72.43	87.28
Factor loadings				
Ag	0.28049824	0.36058523	0.22884688	–0.042919712
Al	–0.06857495	0.20315907	–0.43063114	–0.379488950
As	–0.05121064	–0.05518212	–0.34705801	0.405466724
Cd	–0.30325016	0.04696802	–0.24139784	0.146971960
Co	–0.25628014	–0.39362905	–0.01490130	–0.270990788
Cr	0.35550861	–0.16782308	–0.31146292	0.087988620
Cu	–0.24463488	–0.42969637	0.03129423	0.239449497
Fe	–0.38800631	0.01130980	0.28401777	–0.002918325
Hg	0.19529704	0.02514386	–0.10527404	0.551485455
Mn	0.41142592	–0.10611255	–0.17327520	–0.170903102
Ni	–0.37176084	0.01307610	–0.26669372	–0.088626259
Pb	0.15622500	–0.50341793	–0.16973290	0.040373604
Se	0.20308896	–0.35947861	0.06333031	–0.405176820
Zn	–0.09112794	0.24669975	–0.50944547	–0.150407636

Table S7. Estimated hazard quotient (HQ) and hazard index (HI) of exposure to elements from consumption of Tschanara Tea Garden teas by European consumers.

Element	BA	BK	BV	GK	OA	OK	VW	YK
	HQ							
Ag	6.3×10^{-6}	6.5×10^{-6}	6.1×10^{-6}	6.5×10^{-6}	6.4×10^{-6}	6.3×10^{-6}	6.3×10^{-6}	7.9×10^{-6}
Al	4.0×10^{-2}	5.2×10^{-2}	4.4×10^{-2}	1.2×10^{-1}	2.5×10^{-1}	7.9×10^{-2}	1.0×10^{-1}	2.3×10^{-2}
As	2.2×10^{-4}	2.3×10^{-4}	2.1×10^{-4}	2.1×10^{-4}	2.0×10^{-4}	2.2×10^{-4}	2.0×10^{-4}	1.8×10^{-4}
Cd	3.4×10^{-4}	1.5×10^{-4}	2.6×10^{-4}	1.8×10^{-4}	3.2×10^{-4}	1.9×10^{-4}	9.3×10^{-5}	1.2×10^{-4}
Co	8.5×10^{-6}	1.4×10^{-5}	4.0×10^{-5}	9.1×10^{-6}	1.7×10^{-5}	9.8×10^{-6}	2.4×10^{-5}	7.6×10^{-6}
Cr	5.5×10^{-4}	7.6×10^{-4}	6.9×10^{-4}	7.8×10^{-4}	7.4×10^{-4}	1.0×10^{-3}	5.4×10^{-4}	7.8×10^{-4}
Cu	1.3×10^{-4}	1.2×10^{-4}	1.6×10^{-4}	1.2×10^{-4}	1.1×10^{-4}	1.1×10^{-4}	1.1×10^{-4}	1.1×10^{-4}
Fe	3.7×10^{-4}	2.3×10^{-4}	3.3×10^{-4}	2.4×10^{-4}	2.8×10^{-4}	2.2×10^{-4}	2.9×10^{-4}	2.7×10^{-4}
Hg	8.6×10^{-5}	1.0×10^{-4}	5.7×10^{-5}	1.2×10^{-4}	4.9×10^{-5}	9.0×10^{-5}	1.8×10^{-5}	9.3×10^{-5}
Mn	2.5×10^{-2}	4.2×10^{-2}	3.6×10^{-2}	4.8×10^{-2}	4.6×10^{-2}	7.3×10^{-2}	4.6×10^{-2}	5.2×10^{-2}
Ni	2.4×10^{-3}	2.4×10^{-3}	2.9×10^{-3}	2.3×10^{-3}	3.2×10^{-3}	1.5×10^{-3}	2.0×10^{-3}	1.9×10^{-3}
Pb	3.3×10^{-4}	5.7×10^{-4}	8.2×10^{-4}	6.2×10^{-4}	4.8×10^{-4}	7.5×10^{-4}	4.2×10^{-4}	5.9×10^{-4}
Se	2.9×10^{-5}	2.9×10^{-5}	4.4×10^{-5}	3.6×10^{-5}	3.8×10^{-5}	4.6×10^{-5}	4.2×10^{-5}	4.3×10^{-5}
Zn	$3.6E \times 10^{-4}$	5.3×10^{-4}	3.5×10^{-4}	3.9×10^{-4}	6.1×10^{-4}	3.8×10^{-4}	3.8×10^{-4}	3.1×10^{-4}
	HI							
All	7.0×10^{-2}	9.9×10^{-2}	8.6×10^{-2}	1.7×10^{-1}	3.1×10^{-1}	1.6×10^{-1}	1.5×10^{-1}	7.9×10^{-2}
